# Gene Replacement in Arabidopsis Reveals Manganese Transport as an Ancient Feature of Human, Plant and Cyanobacterial UPF0016 Proteins

**DOI:** 10.3389/fpls.2021.697848

**Published:** 2021-06-14

**Authors:** Natalie Hoecker, Yvonne Hennecke, Simon Schrott, Giada Marino, Sidsel Birkelund Schmidt, Dario Leister, Anja Schneider

**Affiliations:** ^1^Molekularbiologie der Pflanzen (Botanik), Fakultät für Biologie, Ludwig-Maximilians-Universität München, Martinsried, Germany; ^2^Massenspektrometrie von Biomolekülen an der LMU (MSBioLMU), Fakultät für Biologie, Ludwig-Maximilians-Universität München, Martinsried, Germany; ^3^Department of Plant and Environmental Sciences, Faculty of Science, University of Copenhagen, Frederiksberg, Denmark

**Keywords:** PAM71, manganese transporter, *Arabidopsis*, UPF0016 evolution, endosymbiosis, gene replacement, *Synechocystis*

## Abstract

The protein family 0016 (UPF0016) is conserved through evolution, and the few members characterized share a function in Mn^2+^ transport. So far, little is known about the history of these proteins in Eukaryotes. In *Arabidopsis thaliana* five such proteins, comprising four different subcellular localizations including chloroplasts, have been described, whereas non-photosynthetic Eukaryotes have only one. We used a phylogenetic approach to classify the eukaryotic proteins into two subgroups and performed gene-replacement studies to investigate UPF0016 genes of various origins. Replaceability can be scored readily in the Arabidopsis UPF0016 transporter mutant *pam71*, which exhibits a functional deficiency in photosystem II. The N-terminal region of the Arabidopsis PAM71 was used to direct selected proteins to chloroplast membranes. Transgenic *pam71* lines overexpressing the closest plant homolog (*CMT1*), human *TMEM165* or cyanobacterial *MNX* successfully restored photosystem II efficiency, manganese binding to photosystem II complexes and consequently plant growth rate and biomass production. Thus AtCMT1, HsTMEM165, and SynMNX can operate in the thylakoid membrane and substitute for PAM71 in a non-native environment, indicating that the manganese transport function of UPF0016 proteins is an ancient feature of the family. We propose that the two chloroplast-localized UPF0016 proteins, CMT1 and PAM71, in plants originated from the cyanobacterial endosymbiont that gave rise to the organelle.

## Introduction

Cross-species replacement of genes in model organisms is a powerful tool with which to investigate whether the corresponding proteins retain their ancestral functions over a billion years of evolution. There are several examples in which human genes have been shown to supply the functions of their orthologous counterparts in plants (Jha et al., 2018; Huber et al., 2020), and plant genes can frequently be replaced by the cyanobacterial ortholog or vice versa (Savidge et al., 2002; Sattler et al., 2003; Lv et al., 2009; Armbruster et al., 2013; Proctor et al., 2018; Yoon et al., 2019). These examples of pairwise replacements can be expanded further with multiple replacement assays to gain insight into the history and evolution of gene families. Gene duplication and subsequent divergence is a major driver of evolution, and each gene/protein family has its own evolutionary history.

The uncharacterized protein family 0016 (UPF0016), also referred to as the Gcr1-dependent translation factor1 (GDT1) family (Thines et al., 2020), is a highly conserved membrane transporter family with members in many lineages of the tree of life (Demaegd et al., 2014). One prominent member, which is responsible for the alternative family name just mentioned, is the Golgi-localized Gdt1p, originally identified as a Ca^2+^/H^+^ antiporter in *Saccharomyces cerevisiae* (Demaegd et al., 2013; Colinet et al., 2016). A second, the human transmembrane protein 165 (TMEM165), attracted much interest when its involvement in the rare condition known as congenital disorders of glycosylation (CDG) was demonstrated in 2012 (Foulquier et al., 2012; Rosnoblet et al., 2013; Zeevaert et al., 2013; Bammens et al., 2015; Potelle et al., 2016; Schulte Althoff et al., 2016; Krzewinski-Recchi et al., 2017; Morelle et al., 2017). Several studies have hypothesized that TMEM165 is a Golgi-localized Mn^2+^ and/or Ca^2+^ transporter (Demaegd et al., 2013; Potelle et al., 2016; Stribny et al., 2020).

In the model plant *Arabidopsis thaliana*, the chloroplast manganese transporter1 (CMT1) and photosynthesis-affected mutant71 (PAM71) proteins were found to transport Mn^2+^ across the chloroplast envelope and the thylakoid membrane, respectively (Schneider et al., 2016; Eisenhut et al., 2018; Zhang et al., 2018). Two additional members of the family are located in the endoplasmic reticulum (ER) compartment (Hoecker et al., 2020) and a third, pam71-like3 (PML3), found in the Golgi membrane, facilitates Mn^2+^ import under conditions of Mn deficiency (Yang et al., 2021).

It has been shown that, in bacteria, members of UPF0016 are located in the plasma membrane, where their main function is the export of excess Mn^2+^ to prevent Mn toxicity. One prominent example is the manganese exporter A (MneA), a UPF0016 protein found in *Vibrio cholerae* (Fisher et al., 2016; Zeinert et al., 2018). Cyanobacteria, such as Synechocystis, possess additional internal membranes, at which photosynthesis takes place. Here, the manganese exporter (MNX) protein presumably needs to be targeted to both the thylakoid and the plasma membrane in order to perform its physiological function and prevent accumulation of toxic levels of Mn^2+^ in the cytosol (Brandenburg et al., 2017; Gandini et al., 2017; Eisenhut, 2019).

Eukaryotic proteins of the UPF0016 family share a conserved overall structure, comprising two regions consisting of three transmembrane (TM) domains each, which are connected by an acidic loop, and contain a highly conserved Glu-x-Gly-Asp-(Arg/Lys)-(Ser/Thr) motif in TM1 and TM4, respectively (Demaegd et al., 2014; Hoecker et al., 2017; Thines et al., 2020). This type—with six TM domains—is also found in many prokaryotes, while some others harbor one or two genes coding for a version that consists of only three TM domains, which are assumed to form homo- or heterodimers (Demaegd et al., 2014). Besides the high degree of conservation, eukaryotic representatives of the UPF0016 family exhibit additional features, among them high sequence diversity within their N-terminal segments (Hoecker et al., 2017). While the ER-localized proteins lack an N-terminal extension, ScGdt1p, HsTMEM165 and AtPML3 all have an N-terminal signal peptide that directs them to the Golgi membrane (Foulquier et al., 2012; Demaegd et al., 2013; Hoecker et al., 2020; Yang et al., 2021). The chloroplast-targeting peptide (cTP) sequences of Arabidopsis PAM71 and CMT1 direct these proteins to the thylakoid and inner-envelope membranes, respectively (Schneider et al., 2016; Eisenhut et al., 2018; Zhang et al., 2018).

In this study, we set out to define the ancestor(s) of eukaryotic members of the UPF0016 family and determine whether individual members have retained the ancestral function. We performed a phylogenetic analysis to identify appropriate candidates. We selected MNX of Synechocystis (Brandenburg et al., 2017; Gandini et al., 2017), human TMEM165 and Arabidopsis CMT1 (Eisenhut et al., 2018; Zhang et al., 2018) and directed the proteins to the thylakoid membrane of the Arabidopsis *pam71* knock-out line. Appropriate control constructs were generated and transgenic *pam71* plants were analyzed for complementation of growth deficiencies as a measure of the degree to which these transporters have retained the ancestral mode of action.

## Materials and Methods

### Phylogenetic Analysis

Protein sequences were retrieved by protein Blast searches at NCBI^1^ using *Arabidopsis thaliana* PAM71 or *Vibrio cholera* MneA as query sequences. Target organisms either represent ten eukaryotic model species or were manually selected from a list of 393 prokaryotes (Hanikenne and Baurain, 2013). Evolutionary analyses were conducted in MEGA X (Kumar et al., 2018) using the maximum-likelihood method and the Le_Gascuel_2008 model (Le and Gascuel, 2008). Initial tree(s) were obtained automatically by first applying Neighbor-Join and BioNJ algorithms to a matrix of pairwise distances estimated using a JTT model, and then selecting the topology with the superior log likelihood value. A discrete Gamma distribution was used to model evolutionary rate differences among sites (+*G*). Estimates of evolutionary divergence between sequences were obtained by using a pairwise distance calculation that assumes uniform rates among sites. All positions with less than 95% site coverage were eliminated; i.e., fewer than 5% alignment gaps, missing data, and ambiguous bases were allowed at any position (partial deletion option).

### Generation of Arabidopsis Lines

The mutants *pam71* or *cmt1* were stably transformed by the floral-dip method (Clough and Bent, 1998) using Silwet L-77 (Lehle Seeds, Round Rock, TX, United States) as the detergent. Both mutations are in the Colombia-0 background. The first step employed primer pairs 1/2 or 16/17 (see Supplementary Table 1), and first-strand cDNA of Arabidopsis for cloning of the cTP sequences into the pENTR vector (Thermo-Fisher Scientific/Invitrogen, Waltham, MA, United States), yielding pENTR-cTP_(PAM71)_ and pENTR-cTP_(CMT1)_. The Phusion High-Fidelity DNA polymerase (New England Biolabs, Frankfurt, Germany) was used for this and all subsequent steps, and all further primer sequences are listed in Supplementary Table 1. The plasmids pENTRcTP_(PAM71)_ and pENTR-PAM71 were used as templates for the amplification of two PCR products using primers 1/2 and primers 3/4, respectively. The two PCR products were then digested with *Xho*I (New England Biolabs), ligated with T4 DNA ligase (New England Biolabs), and a second PCR was performed using primers 1/4. The resulting PCR product was cloned into pENTR, yielding the plasmid pENTR-cTP_(PAM71)_:PAM71_*core*_. The plasmid pENTR-cTP_(PAM71)_:CMT1_*core*_ was assembled in an analogous manner, using primers 1/2 with pENTRcTP_(PAM71)_ and primers 6/7 with pENTR-CMT1 as templates for the first PCR, and primers 1/7 for the second PCR. The plasmid pENTR-cTP_(PAM71)_:TMEM165 was assembled in the same way, using primers 1/2 with pENTRcTP_(PAM71)_ and primers 8/9 with pDNR-LIP-TMEM165 (transOMIC technologies, Huntsville, AL, United States) as templates for the first PCR and primers 1/9 for the second PCR. Plasmid pENTR-PAM71_*core*_ was created using primers 5/4 and pENTR-PAM71 as a template. Plasmids pENTR-cTP_(CMT1)_:CMT1core and pENTR-cTP_(CMT1)_:PAM71 were constructed accordingly. All plasmids were sequenced and recombined into the destination vector pB2GW7 (Karimi et al., 2002) using the LR Clonase Gateway system (Thermo-Fisher Scientific/Invitrogen). Plasmid pB2GW7p35S:cTP_*extended(PAM71)*_:MNX was assembled using three PCR products and the Gibson assembly cloning kit (New England Biolabs). The three PCR products were obtained with primer pairs 10/11, 12/13, and 14/15, and pENTR-PAM71, *Synechocystis sp. PCC6803* genomic DNA (Gandini et al., 2017) and pB2GW7, respectively, as templates. The final construct was then sequenced. All plasmids harboring the pB2GW7 backbone were transformed into *Agrobacterium* and plated on media containing Rif/Gent/Spec. Individual colonies were selected, verified and used to transform Arabidopsis. Transgenic T_1_ plants were selected by spraying seedlings twice with BASTA at a final concentration of 100 mg L^–1^ glufosinate-ammonium. T_2_ plants were assayed for segregation by genotyping, and appropriate homozygous T_3_ seed stocks were generated.

### Growth Conditions

Arabidopsis seeds were stratified for 3 days at 4°C to synchronize germination, and plants were grown for 4–5 weeks in growth chambers on a 12–12 h light-dark cycle at 18–22°C and 100 μmol photons m^–2^ s^–1^ during the light period. The Arabidopsis plants used for transformation and seed production, and *Nicotiana benthamiana* plants were grown in a temperature-controlled greenhouse with additional lighting for up to 16 h to reach a fluence of at least 140 μmol photons m^–2^ s^–1^.

Plant growth was analyzed on the basis of leaf area, which was determined from photographs taken at different times after germination and quantified using the ImageJ software (Schneider et al., 2012).

### Transient Expression

The plasmids pENTR-cTP_(PAM71)_ and pENTR-cTP_(CMT1)_ were recombined into the destination vector pB7FWG2 (Karimi et al., 2002), resulting in pB7FWG2p35S:cTP_(PAM71)_:GFP and pB7FWG2p35S:cTP_(CMT1)_:GFP, respectively, which were then transformed into *Agrobacterium*. *Agrobacterium*-mediated infiltration of 4-week-old *N. benthamiana* leaves and protoplast isolation were performed as described (Schweiger and Schwenkert, 2014). In brief, 48 h after infiltration, protoplasts were released from leaf tissue by incubation in the dark for 4 h, with mild shaking at 40 rpm, in an enzyme solution (osmolarity: 550 mOsm) containing 1% (w/v) cellulase (Duchefa Biochemie, Haarlem, Netherlands) and 0.3% (w/v) macerozyme (Duchefa Biochemie). The protoplasts were then filtered through a 100 μm gauze filter and washed with W5 buffer [150 mM NaCl, 125 mM CaCl_2_, 5 mM KCl, 2 mM MES (pH 5.7), 550–580 mOsm], then centrifuged at 100 *g* for 1 min and resuspended in W5 buffer. The fluorescence signals were recorded with a confocal laser-scanning microscope (Leica microsystems TCS SP5, Leica Microsystems, Wetzlar, Germany) with a 63x objective. The argon laser was set to 30%, GFP fluorescence was excited at 488 nm and the emission was recorded with a PMT detector at 515 nm. Chlorophyll autofluorescence was detected at 650–705 nm with a second PMT detector. The gain settings for both PMT detectors were chosen within the interval 760–900 to reduce background noise. Z-stacking images were generated with a maximum of 0.5 μm distance between each layer.

### Chlorophyll *a* Fluorescence Measurements

The photosynthetic performance of PSII was quantified by measuring chlorophyll *a* fluorescence using a Dual PAM fluorometer (Walz, Effeltrich, Germany). Plants were dark-adapted for 30 min and single leaves were exposed to probe light (3–4 μmol photons m^–2^ s^–1^) for 30 s to measure minimal chlorophyll *a* fluorescence (F_0_). A light pulse of 10,000 μmol photons m^–2^ s^–1^ was then applied for 800 ms to determine the maximum chlorophyll *a* fluorescence (Fm). Quantitative information was obtained by calculating the maximum quantum yield of PSII as Fv/Fm = (Fm-F_0_)/Fm (Maxwell and Johnson, 2000). *In vivo* chlorophyll *a* fluorescence was captured as a photograph by the Imaging PAM fluorometer (Walz, Effeltrich, Germany). Plants were dark-adapted for 30 min and whole plants exposed to blue probe light (3–4 μmol photons m^–2^ s^–1^) for 30 s, followed by a light pulse of 2,800 μmol photons m^–2^ s^–1^ for 800 ms.

### Real-Time PCR Analysis

Total RNA was isolated from Arabidopsis leaves using the Monarch Total RNA Miniprep Kit including the on-column DNase digest, and cDNA was synthesized according to the instructions supplied with the iScript cDNA Synthesis Kit (Bio-Rad, Hercules, CA, United States). For quantitative real-time (qRT) PCR, SYBR Green Supermix (Bio-Rad) was used and PCR was performed with the CFX light cycler (Bio-Rad). Selected regions of the *cTP*_(PAM71)_ sequence (primers 30/31) or the *PAM71*_(core)_ sequence (primers 32/33) were used to record expression of the target gene and the reference gene *Actin* (primers 28/29). Relative transcript levels were quantified according to the comparative cycle threshold (C_*T*_) method (Schmittgen and Livak, 2008): ΔC_*T*_ = C_*T*_ (Gene_*TransgenicLine*_) − C_*T*_ (Actin_*TransgenicLine*_) or ΔC_*T*_ = C_*T*_ (Gene_*Col–0*_) − C_*T*_ (Actin_*Col–0*_); ΔΔC_*T*_ (TransgenicLine) = ΔC_*T*_ (Gene_*TransgenicLine*_) − ΔC_*T*_ (Gene_*Col–0*_) and 2^–ΔΔ*CT*^ calculated. Expression in Col-0 is: ΔΔC_*T*_ (Col-0) = ΔC_*T*_ (Gene_*Col–0*_) − ΔC_*T*_ (Gene_*Col–0*_) = 0 and 2^0^ = 1.

### Blue-Native PAGE and SEC-ICP-MS Analysis

For the isolation of thylakoid membrane-protein complexes, leaf samples (5 g fresh weight) were homogenized in 0.4 M sucrose, 10 mM NaCl, 5 mM MgCl_2_, 20 mM Tricine (pH 7.9), 10 mM ascorbate and 10 mM NaF, then filtered through two layers of Miracloth (GE Healthcare, Chicago, IL, United States) and centrifuged at 5,000 *g* for 10 min at 4°C. The pellet was dissolved in 5 mM Tricine (pH 7.9) and 10 mM NaF, and centrifuged at 10,000 *g* for 10 min at 4°C. The resulting thylakoid membranes were dissolved in storage buffer [0.4 M sucrose, 10 mM NaCl, 5 mM MgCl_2_, 20 mM Tricine (pH 7.9), 20% (v/v) glycerol, 10 mM NaF] and the protein concentration was determined using the Pierce BCA Protein Assay (Thermo-Fisher Scientific/Invitrogen, Waltham, MA, United States). Samples were stored at −80°C. For Blue-Native PAGE analysis, samples equivalent to 18 μg protein were solubilized in the presence of 1% (w/v) β-dodecyl-maltoside (Sigma-Aldrich, St. Louis, MO, United States) for 10 min on ice in the dark. Native-PAGE sample buffer was then added and protein complexes were separated by non-denaturing Bis-Tris gel electrophoresis (3–12% acrylamide gradient, Thermo-Fisher Scientific/Invitrogen) for 3.5 h. For SEC-ICP-MS analysis, samples equivalent to 50 μg protein were solubilized in the presence of 1% (w/v) α-dodecyl-maltoside (Biomol, Hamburg, Germany) for 10 min on ice in the dark. Solubilized protein complexes were filtered through a 0.45-μm nylon membrane and applied to a size-exclusion column (BioBasic SEC1000, a silica-based high-pressure steel column; Thermo-Fisher Scientific/Invitrogen) using an inert HPLC system. Protein elution was performed with 25 mM Bis-Tris (pH 7.0) and 0.03% (w/v) α-dodecyl-maltoside as the mobile phase at a flow rate of 1 mL min^–1^. The outlet of the column was coupled to a triple quadrupole ICP-MS (Agilent 8800 ICP-QQQ-MS, Agilent, Santa Clara, CA, United States) for online detection of Mn binding in the size-fractionated PSII complexes. The ICP-QQQ-MS was operated in MS/MS scan mode with oxygen as the reaction gas, which allowed for simultaneous detection of manganese as the parent ion (^55^Mn^+^) and sulfur as the oxide product ion (^48^SO^+^). The external calibration for quantification of the stoichiometric ratios of Mn to S in photosynthetic complexes was carried out using flow-injection analysis (Schmidt et al., 2015).

### Proteome Analysis

For the isolation of chloroplasts, leaf samples (12 g fresh weight) were homogenized in a mixture of 0.4 M sorbitol, 20 mM Tricine-NaOH (pH 8.4), 10 mM EDTA, 0.1% (w/v) BSA, 5 mM NaHCO_3_, 1 mM MgCl_2_ and 1 mM MnCl_2_. The extract was filtered through two layers of Miracloth (GE Healthcare) and concentrated by centrifugation for 5 min at 1,500 *g*. The pellet was resuspended in 80 mM sorbitol, 4 mM Tricine-NaOH (pH 8.4), 0.5 mM EDTA, 1 mM MgCl_2_ and then layered onto a discontinuous 40% (w/v)/80% (w/v) Percoll gradient (GE Healthcare). Intact chloroplasts were isolated from the interphase after centrifugation for 15 min at 7,000 *g*. To separate envelope and thylakoid membranes, the chloroplasts were lysed in 10 mM HEPES-KOH (pH 7.6) and 5 mM MgCl_2_ by incubation on ice for 10 min, then layered onto a discontinuous sucrose gradient consisting of 1.2 M, 1.0 M, and 0.46 M sucrose, and centrifuged at 58,000 *g* for 2 h. The stroma in the top fraction was discarded, the envelopes were collected from the middle fraction, and the thylakoids were recovered as a pellet. The envelope fraction was further concentrated by centrifugation at 135,200 *g* for 1 h at 4°C. Both fractions were stored in TMK buffer [10 mM Tris–HCl pH 6.8, 10 mM MgCl_2_, 20 mM KCl including protease inhibitor cocktail (Roche, Basel, Switzerland)] at −80°C.

Thylakoid and envelope fractions were resuspended in 200-μl aliquots of guanidine-HCl, and incubated at 60°C for 30 min. Samples were then disrupted by applying two 10-s bursts of sonication using a Branson Sonifier B-12 (Branson Ultrasonics, Brookfield, CT, United States). After removing cell debris by centrifugation at 10,000 *g* for 15 min, samples were loaded onto a Microcon-30 kDa filter. On-column reduction, alkylation and tryptic digestion were performed as previously described (Janowski et al., 2018). After elution, the peptide mixtures were acidified to pH ≤ 3 using formic acid, desalted with home-made C18 stage tips (Rappsilber et al., 2003), vacuum dried to near dryness and stored at −80°C until further use.

LC-MS/MS was performed on an Ultimate 3000 RSLCnano HPLC system (Thermo-Fisher Scientific/Invitrogen), coupled online to an Impact II Ultra-High Resolution Qq-Time-Of-Flight (Bruker Daltonics, Billerica, MA, United States). The nano-LC system (Thermo-Fisher Scientific/Invitrogen) was operated in a two-column setup with an Acclaim Pepmap nano-trap column (C18, 100 Å, 100 μm × 2 cm) and an Acclaim Pepmap RSLC analytical column (C18, 100 Å, 75 μm × 50 cm). The columns were kept at 50°C throughout the run. The peptide mixtures were separated at a constant flow rate of 250 nl/min, using a linear gradient 3–30% solvent B (0.1% formic acid in acetonitrile) over 60 min, followed by a linear increase from 30 to 45% solvent B within 15 min. Full MS scans in the mass range 200–2,000 m/z were acquired at a rate of 3 Hz, and the 18 most intense peaks were selected for MS2 analysis using an intensity-dependent spectrum acquisition rate of between 4 and 16 Hz. To minimize repeated sequencing, the dynamic exclusion duration was set to 30 s.

The raw MS files were processed using the MaxQuant software (Cox and Mann, 2008). Peak lists were searched against the Arabidopsis reference proteome (Uniprot, version April 2019) using the built-in Andromeda search engine (Cox et al., 2011). The database was modified to include the sequences of the chimeric constructs. The search parameters were set as follows: Cysteine carbamidomethylation as fixed modification, methionine oxidation and acetylation of protein N-termini as variable modifications. The specific protease was set to trypsin (Thermo-Fisher Scientific/Invitrogen), allowing a maximum of two missed cleavage sites. The false-discovery rate at the protein and PSM level was set to 1%. The match-between-runs option was disabled, whereas the intensity-based-absolute-quantification (iBAQ) (Schwanhausser et al., 2011) option was enabled. Downstream data analysis was performed using Microsoft Excel. Potential contaminants, reverse hits and proteins identified only by site modification were removed. The protein group iBAQ values were then normalized to the sum of all iBAQ values within one sample, generating relative iBAQ (riBAQ) values. To compare the thylakoid and envelope fractions of single chloroplast preparations, the abundances of a given protein group were expressed as relative percentages of the total riBAQ values. Data were analyzed from three independent biological replicates.

### Data and Code Availability

The mass spectrometry proteomics data have been deposited with the ProteomeXchange consortium http://proteomecentral.proteomexchange.org *via* the PRIDE partner repository (Vizcaino et al., 2016) under the accession number PXD022763.

## Results

### Phylogenetic Analysis of UPF0016 Proteins

**FIGURE 1:**
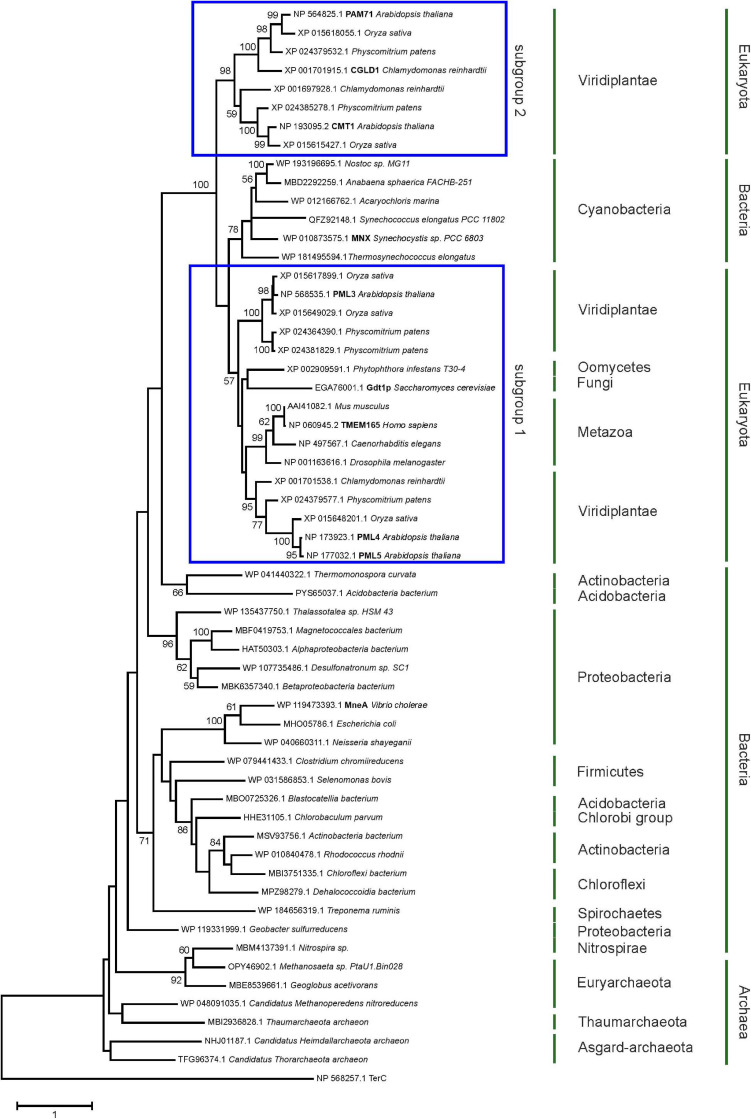
Evolutionary analysis of UPF0016 members using the maximum-likelihood method. Protein sequences were retrieved by protein BLAST search using AtPAM71 as query sequence, except for those from *Alphaproteobacterium bacterium*, *Vibrio cholera*, *Escherichia coli, Neisseria shayeganii*, and *Treponema ruminis*, which were identified using MneA of *Vibrio cholera* as query sequence. The tree with the highest log likelihood is shown. The percentage (>50%) of trees in which the taxa grouped together is also shown (bootstrap values based on 500 replicates). The tree is drawn to scale with branch lengths measured in numbers of substitutions per site. The tree is rooted on *Arabidopsis thaliana* TerC. The TerC and UPF0016 families form part of the LysE transporter superfamily (http://pfam.xfam.org/clan/CL0292). NCBI accession numbers are indicated and proteins mentioned throughout the text are highlighted.

**FIGURE 2:**
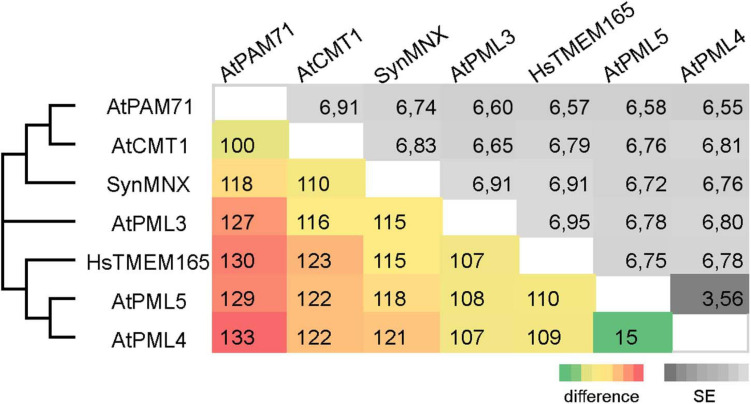
Pairwise comparison of UPF0016 members of Arabidopsis, Synechocystis and human. The numbers of amino-acid differences between the indicated sequences are shown. Standard-error (SE) estimates are shown above the diagonal and were obtained by a bootstrap procedure (1,000 replicates). The consensus tree in which the associated proteins clustered together is indicated on the left.

The family was first described in 2014 (Demaegd et al., 2014) and members have now been identified in more than 2,800 species across all three domains of life (Supplementary Figure 1). We were particularly interested in examining the ancestry of plant UPF0016 proteins, which are encoded by small gene families (Hoecker et al., 2017). In order to embed plant UPF0016 proteins in a broader context, proteins of prokaryotic and eukaryotic origins were retrieved and subjected to a phylogenetic analysis. A dataset of 33 proteins was selected from 33 prokaryotic organisms, which were chosen from a taxonomically representative set of phyla (Hanikenne and Baurain, 2013), taking into account phyla that appear to encompass many species that code for UPF0016 proteins (Supplementary Figure 1). This set was supplemented by 24 eukaryotic proteins obtained from 10 species, representing both photosynthetic and non-photosynthetic model organisms. Clear phylogenies could not be identified within prokaryotic phyla, except for those proteins of α-Proteobacteria that displayed a bootstrap value of 100%, indicating that these could be monophyletic (Figure 1). Intriguingly, cyanobacterial proteins were found to be closely related to eukaryotic proteins (as indicated by a single branch supported by a bootstrap value of 100%) and were clearly separated from all other prokaryotic phyla. Closer inspection of the members of Viridiplantae confirmed the previous findings (Hoecker et al., 2017) that chloroplast-localized members and Golgi/ER-localized members form separate subgroups (Figure 1). The latter subgroup comprises members found in all Eukaryotes, including Metazoa and Fungi, and was named subgroup one. The chloroplast-localized proteins were named subgroup two. These proteins presumably arose separately, because they form a single defined monophyletic group. Next, we extracted the well characterized proteins of Arabidopsis, Synechocystis and human, and estimated the evolutionary divergence between these seven sequences by pairwise comparisons. In terms of their amino-acid sequences, PAM71 and AtPML4 are most dissimilar, while AtPML4 and AtPML5 display the highest similarity (Figure 2). HsTMEM165 shows less resemblance to SynMNX, AtPAM71, and AtCMT1 than to the three AtPML proteins, or in other words SynMNX, AtPAM71, and AtCMT1 form a cluster. Taken together, these observations allow us to conclude that the gene(s) encoding the chloroplast-localized proteins PAM71 and CMT1 are derived from the cyanobacterial endosymbiosis that gave rise to chloroplasts. Because the gene(s) encoding Golgi/ER-localized proteins have close counterparts in non-photosynthetic Eukaryotes, this clade must have arisen prior to the cyanobacterial endosymbiosis.

### The Arabidopsis Mutant *pam71* as a Platform for UPF0016 Gene-Replacement Studies

**FIGURE 3:**
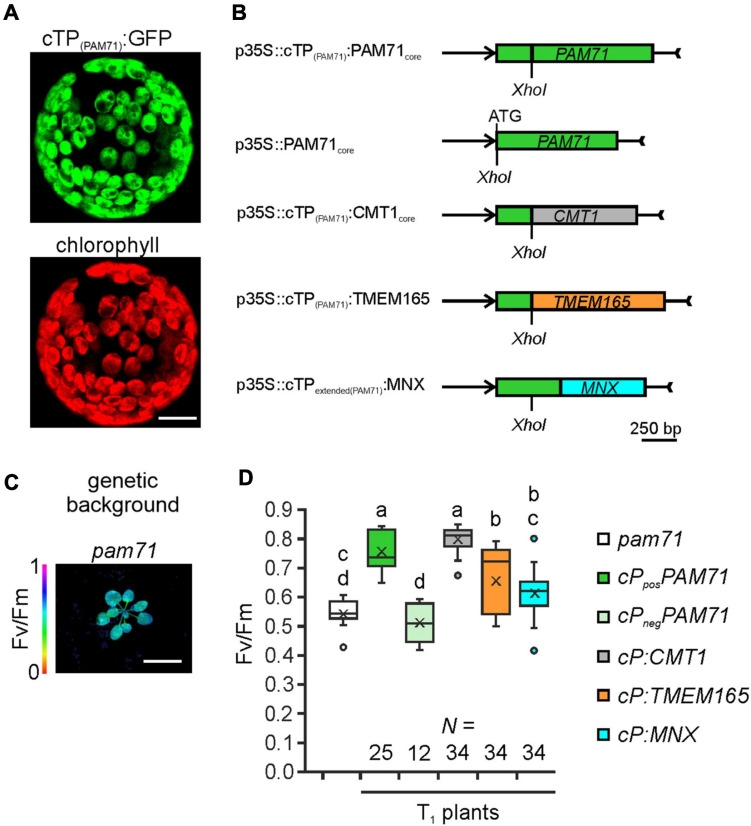
Experimental setup for gene replacement assays. **(A)** Verification of the N-terminal PAM71 sequence as a chloroplast-targeting peptide. *Nicotiana benthamiana* leaves were infiltrated with the *Agrobacterium* strain containing p35S:cTP_(PAM71)_:GFP; protoplasts were isolated after 48 h and analyzed by confocal microscopy. Scale bar = 12.5 μm. **(B)** Scheme depicting the chimeric constructs used for transformation of *pam71.* **(C)** The *pam71* mutant as genetic background. The maximum quantum yield of PSII (Fv/Fm) of a 4-week-old plant is shown. Scale bar = 1 cm. **(D)** Photosynthetic activity of BASTA-resistant T_1_ transgenic plants in comparison to *pam71*. The maximum quantum yields of PSII (Fv/Fm) of T_1_ plants are depicted as box plots representing the range of values, the exclusive median and the mean, indicated as x. The number of independent individuals is given (*N*). Outliers are indicated as dots. Different letters indicate statistical significance based on ANOVA (*p* < 0.01, Tukey’s honestly significant difference (HSD) test).

To test the hypothesis that UPF0016 proteins share an ancient functionality, we chose the *pam71* Arabidopsis mutant for a gene-replacement study. We selected AtCMT1 and HsTMEM165 as representative members of each subgroup and SynMNX as the cyanobacterial representative. The nucleus-encoded PAM71 protein is equipped with a chloroplast-targeting signal peptide at the N-terminus, which is predicted to be cleaved at amino acid position 74 (Schneider et al., 2016; Hoecker et al., 2017) during uptake into the organelle from the cytoplasm. To verify that the cTP is sufficient to direct proteins of interest into this plant organelle, we cloned the first 255 bp of the *PAM71* ORF upstream of the *GFP* sequence and transiently expressed the fusion protein in *Nicotiana benthamiana* leaves (Figure 3A). Indeed, the GFP fluorescence signal was found to coincide precisely with the red autofluorescence emitted by chlorophyll *a* molecules in isolated protoplasts. Next, a series of chimeric constructs was generated (Figure 3B), including a positive control containing the native *PAM71* sequence and a negative control containing a truncated version of *PAM71* lacking the first 255 bp at the 5′ end. Furthermore, we linked the *PAM71 cTP* sequence to the full-length *TMEM165* sequence and replaced the original *cTP* sequence of *CMT1*. An extended *cTP* sequence was used to generate the *MNX* construct, in order to increase the size of the protein (Supplementary Figure 2), as the naturally occurring MNX/SynPAM71 is smaller than any of the other three proteins (Thines et al., 2020). The five constructs were introduced into *Agrobacterium* cells and successfully transformed into the *pam71* background. The *pam71* mutant is characterized by a reduced growth rate and a lower maximum quantum yield of photosystem II (PSII) (Fv/Fm, Figures 3C,D) (Schneider et al., 2016; Wang et al., 2016). Several independent transgenic lines per construct were selected based on their resistance to glufosinate, assayed for the presence of the transgene in the homozygous *pam71* background and characterized with respect to their Fv/Fm values. Transgenic lines were named as follows: *cP* refers to *cTP*_*PAM71*_ and *pos* and *neg* indicate presence or absence of *cTP*_*PAM71*_ respectively, followed by the protein name. Fv/Fm was significantly reduced in the twelve *cP_*neg*_PAM71* lines relative to the twenty-five *cP_*pos*_PAM71* control lines (Figure 3D), indicating that the presence of a cTP is required for functional complementation. Interestingly, the Fv/Fm values of the thirty-four selected *cP:CMT1* lines were comparable to those of the positive control lines (Figure 3D). However, it is worth noting that the endogenous proteins PAM71 and CMT1 have non-redundant functions in chloroplasts (Eisenhut et al., 2018; Zhang et al., 2018; Frank et al., 2019; Schmidt et al., 2020). Moreover, it was found that the thirty-four *cP:TMEM165* lines and the thirty-four selected *cP:MNX* lines have significantly increased Fv/Fm values in comparison to the negative control lines and *cP:TMEM165* lines are significantly improved in comparison to *pam71* (Figure 3D). Both groups (*cP:TMEM165* lines and *cP:MNX* lines) showed a wide range of Fv/Fm values, for instance from 0.4 to 0.8 in the *cP:MNX* lines, maybe due to very different levels of expression of the respective non-plant transgene across the populations. Taken together, these results suggested that the approach is feasible, and we proceeded to analyze individual lines in more detail.

### In Transgenic Lines Both the Quantum Yield of Photosystem II and Growth Rate Are Enhanced

**FIGURE 4:**
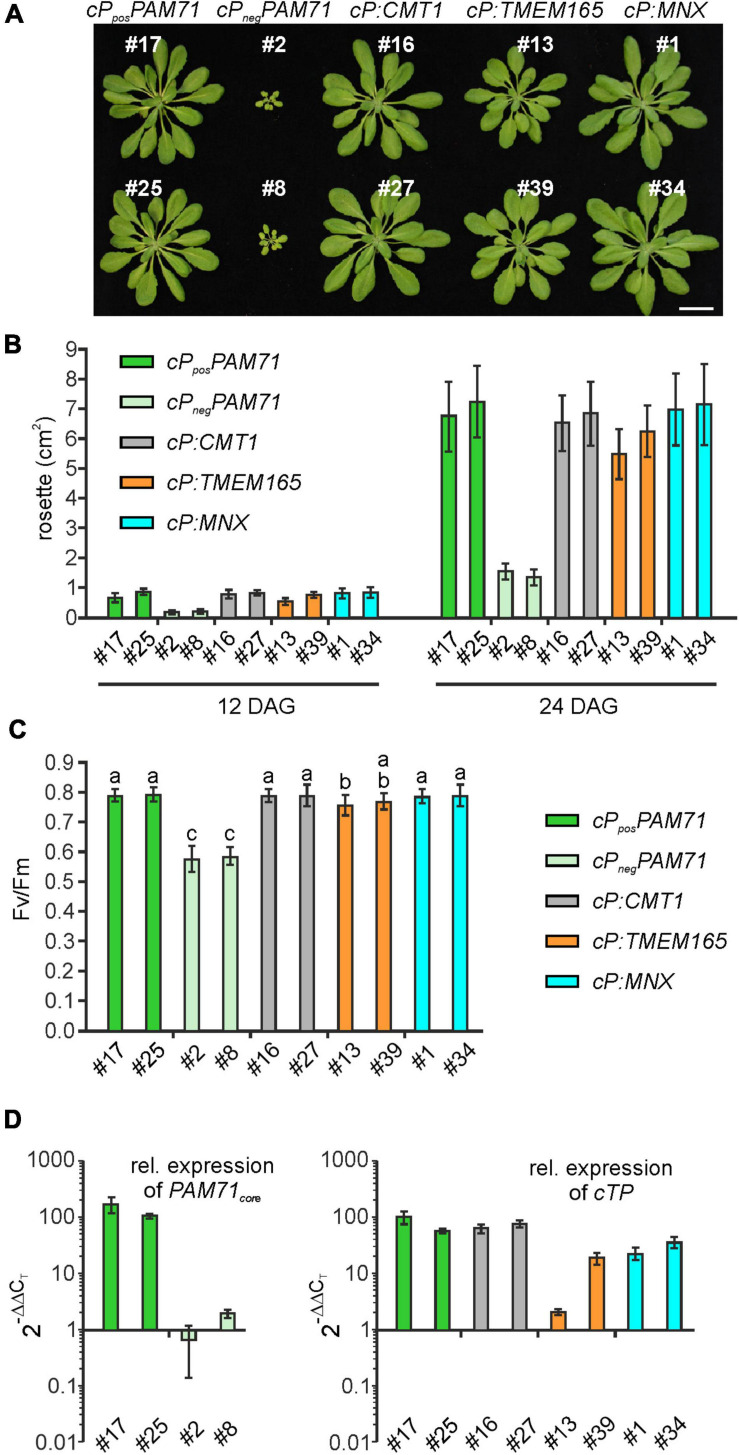
Growth phenotype, photosynthetic activity and expression of transgenes in individual transgenic lines with *pam71* genetic background. **(A)** Rosette phenotype of the indicated transgenic lines. Plants were grown for 5 weeks in a 12–12 h light-dark cycle. Scale bar = 2 cm. **(B)** Quantification of growth phenotypes of the indicated transgenic lines. Leaf areas of the different genotypes were determined from photographs taken 12 days after germination (DAG) and 24 days after germination (DAG). Mean values ± SD (*n* = 10) are shown. **(C)** Photosynthetic activity of the indicated transgenic lines. Maximum quantum yields of PSII (Fv/Fm) are shown as mean values ± SD (*n* ≥ 17). Plants were grown for 4 weeks on a 12–12 h light-dark cycle at 100 μmol photons m^–2^ s^–1^. Different letters indicate statistical significance according to ANOVA (*p* < 0.05, Tukey’s HSD test). **(D)** Expression analysis in transgenic lines. The color code for genotypes is identical to that in panels **(B,C)**. Quantitative real-time PCR (qRT-PCR) was performed using primer combinations qRT_PAM71_fwd/qRT_PAM71_rev (Supplementary Table 1) for expression of *PAM71*_*core*_ and qRT_PAM71cTP_fwd/qRT_PAM71cTP_rev (Supplementary Table 1) for expression of *cTP*_*PAM71*_. Expression levels are relative values based on the expression levels in Col-0 (= 1), and *Actin2* as reference gene. Mean values ± SD are based on three biological replicates.

To select suitable lines for detailed investigations, two criteria were applied: T1 plants should exhibit an increased Fv/Fm value and harbor a single-locus transgene insertion, because this is less likely to cause (post)-transcriptional gene silencing in subsequent generations. We selected two lines that met these criteria from each group (Supplementary Figure 3). Single-locus insertion events were identified based on a segregation analysis of T2 plants, assuming a 3:1 mode of dominant inheritance of the transgene (Supplementary Figure 3). Furthermore, we confirmed that the phenotypes of positive *cP_*pos*_PAM71* and negative *cP_*neg*_PAM71* control plants were close to wild-type and *pam71* plants, respectively (Supplementary Figure 4A). All selected transgenic lines were grown side by side, and on visual inspection just before the transition to the reproductive state, the rosette diameter was found to be larger in all cases than in either of the negative control lines (Figure 4A). Growth recovery to almost control levels was observed in the *cP:CMT1*, *cP:TMEM165*, and *cP:MNX* lines (Figure 4B). Furthermore, Fv/Fm values in both *cP_*pos*_PAM71* lines reached almost 0.8, indicative of optimal PSII efficiency and photosynthesis, while both *cP_*neg*_PAM71* lines had Fv/Fm values around 0.6 (Figure 4C). These results are similar to those of wild-type and *pam71* plants respectively (Supplementary Figure 4B). Optimal PSII efficiency was also observed in the two *cP:CMT1* lines, in one of the *cP:TMEM165* lines (#39) and in both *cP:MNX* lines (Figure 4C). The second *cP:TMEM165* line #13 did not quite reach the control value (Figure 4C), but this can probably be explained by variation in the levels of transgene expression. Based on wild-type *cTP* expression, in fact, an approximately twofold higher transgene expression was observed in *cP:TMEM165* #13 (Figure 4D), whereas transgene expression was increased by at least 10-fold in the two positive control lines, in *cP:CMT1* #16 and #27, in *cP*:*TMEM165* #39, and in *cP:MNX* #1 and #34 (Figure 4D). Notably, we also found transgene expression in the negative control lines to be well above the *pam71* level, which can be attributed to the use of the 35S promoter (Supplementary Figure 4C). Overall, we concluded that transgene overexpression in the *cP_*pos*_PAM71, cP:CMT1, cP:MNX* and *cP:TMEM165* lines resulted in the restoration of optimal PSII efficiency, and consequently boosted plant growth and biomass production. It is worth pointing out here that no leaf abnormalities were detected in any of the lines (Figure 4A). In contrast, overexpression of the UPF0016 transporter *PML3* in the Golgi apparatus of Arabidopsis was accompanied by stunted plant growth and a curled leaf morphology (Hoecker et al., 2020).

### Human TMEM165, Plant CMT1, and Cyanobacteria MNX All Facilitate Incorporation of Manganese Into the Photosynthetic Complexes in the Thylakoid Membrane

**FIGURE 5:**
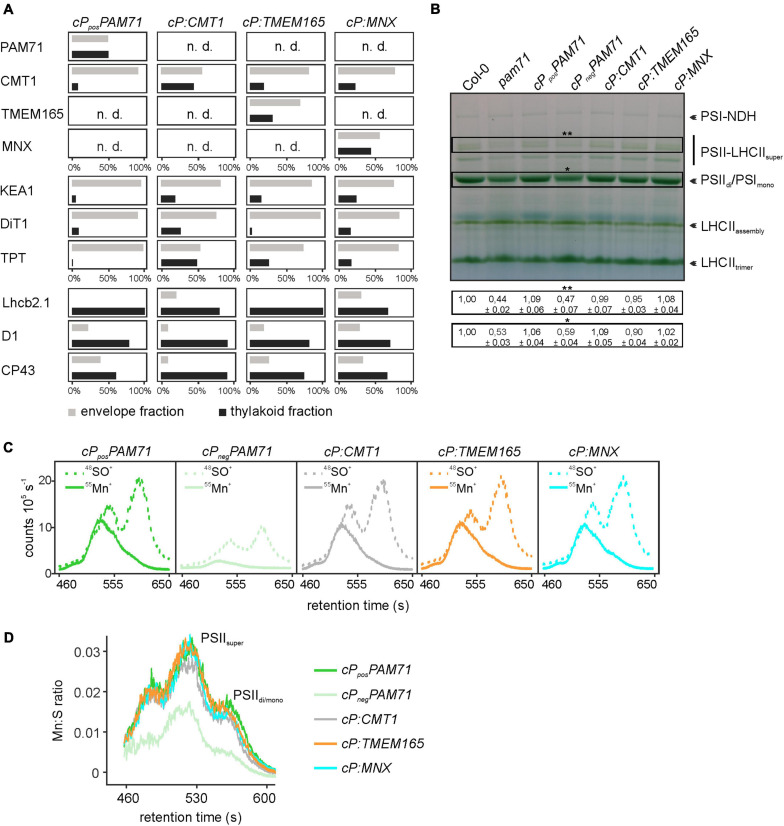
Proteomic and ICP-MS analysis of individual transgenic lines with *pam71* genetic background. **(A)** PAM71, CMT1, TMEM165, MNX and marker proteins of envelope and thylakoid enriched fractions of the indicated plant lines. The K + efflux antiporter 1 (KEA1), dicarboxylate transporter 1 (DiT1) and triosephosphate/phosphate translocator (TPT) were used as envelope marker proteins, and chlorophyll a-b binding protein 2.1 (Lhcb2.1), photosystem II protein D1 (D1) and photosystem II CP43 reaction center protein (CP43) served as thylakoid markers. The proteomic analysis is based on three independent experiments and data depicted are derived from Experiment 2 (Supplementary Tables 3–6). n.d., not detected. **(B)** Accumulation of thylakoid photosynthetic complexes in transgenic lines in comparison to levels in Col-0 and *pam71*. Thylakoid membrane samples (equivalent to 18 μg of protein) were solubilized with 1% (w/v) β-dodecyl-maltoside, and protein complexes were fractionated by Blue-Native gel electrophoreses. NDH, NADH dehydrogenase-like; PSI, photosystem I; PSII, photosystem II; LHCII, light-harvesting complex of photosystem II. The different assembly states are indicated. Quantification of the indicated complexes (**PSII-LHCII supercomplexes and *PSII dimer/PSI monomer complexes) is based on intensity levels in Col-0 (=1,00). Mean values ± SD (*n* = 3) are shown. **(C)** Size-exclusion chromatograms depicting the relative intensities of the non-oxide ion ^55^Mn^+^ and the oxide ion ^48^SO^+^ in the transgenic lines. Thylakoid membranes (equivalent to 50 μg of protein) were solubilized with 1% (w/v) α-dodecyl-maltoside, and protein complexes were fractionated on a size-exclusion column. Analysis was performed with two independent experiments. **(D)** Quantification of the Mn:S ratios in the transgenic lines. Mn:S ratios are shown for the fractionated photosynthetic complexes from the samples analyzed in panel **(C)**. PSII, Photosystem II; different assembly states are indicated. The data are based on two independent experiments.

To unequivocally demonstrate the subcellular localization of cP:TMEM165, cP:CMT1 and cP:MNX in the *pam71* mutant background, proteomic analysis was employed. In this approach, envelope and thylakoid membrane fractions were isolated from purified chloroplasts, and several membrane proteins with known localizations were chosen as marker proteins for quality control. All selected marker proteins could be assigned to the correct fraction in wild-type chloroplasts (Supplementary Table 2), including the endogenous CMT1 protein, whereas the endogenous PAM71 was not detectable. We also verified that PAM71, TMEM165 and MNX were all undetectable in the negative control line, and only the endogenous CMT1 protein was found to be enriched in the envelope fraction (Supplementary Table 2). Chloroplasts were isolated from *cP_*pos*_PAM71* #25, *cP:CMT1* #27, *cP:TMEM165* #39, and *cP:MNX* #1, and the envelope and thylakoid fractions were analyzed for the presence of the respective test proteins and marker proteins (Figure 5A). PAM71 was found to be equally enriched in both fractions in *cP_*pos*_PAM71* plants, as was the MNX protein in *cP:MNX* plants (Figure 5A and Supplementary Tables 3, 4). The majority of the TMEM165 protein was found in the envelope fraction in *cP:TMEM165* plants, but a small portion was also present in the thylakoid membrane (Figure 5A and Supplementary Table 5). Owing to the presence of the endogenous CMT1 protein, cP-CMT1 is difficult to quantify accurately, but nearly 40% of all CMT1 could be assigned to the thylakoid fraction of *cP:CMT1* plants (Figure 5A and Supplementary Tables 6, 7). This result differs from the distribution of endogenous CMT1 in the other genotypes, which had about 19% in their thylakoid enriched fractions (Supplementary Table 7). Thus, we concluded that overexpression of *cP:TMEM165*, *cP:MNX* or *cP:CMT1* in the *pam71* mutant background enables the production of significant amounts of the corresponding proteins, which were eventually inserted into the thylakoid membrane.

One physiological consequence of the *PAM71* knock-out mutation is a marked decrease in the amounts of photosynthetic complexes, for instance PSII dimer, PSI monomer and PSII LHCII supercomplexes (Schneider et al., 2016; Wang et al., 2016). Thus, we tested the ability of *cP:CMT1*, *cP:TMEM165* and *cP:MNX* plants to enhance levels of photosynthetic membrane complexes. To this end, we employed Blue-Native PAGE analysis to determine the amounts of these complexes in the transgenic lines relative to wild-type and *pam71*. As expected, the steady-state levels of PSII_*di*_/PSII_*mono*_ and PSII-LHCII_*super*_ were reduced in the negative control line (*cP_*neg*_PAM71*) to quantities comparable to those in *pam71* (Figure 5B). On the other hand, steady-state levels of these protein complexes were restored to wild-type amounts in *cP_*pos*_PAM71*, *cP:CMT1*, and *cP:MNX* and almost to wild-type levels in *cP:TMEM165* plants. In the *pam71* mutant, inadequate amounts of Mn are bound in PSII complexes (Schneider et al., 2016) and, as expected, the same effect was observed in the negative control line (Figures 5C,D and Supplementary Figure 4D). In contrast, the levels of Mn bound in PSII complexes in *cP:CMT1*, in *cP:TMEM165* and in *cP:MNX* plants matched those seen in the positive control (Figure 5C), as determined by SEC-ICP-MS analysis. Moreover, the Mn:S ratios (the relative amount of Mn incorporated into PSII per unit of S, used here as a proxy for protein) in these three lines were indistinguishable from that in *cP_*pos*_PAM71* (Figure 5D), indicating sufficient transport of Mn^2+^ into the thylakoid lumen.

### CMT1 of Arabidopsis Cannot Be Replaced by PAM71

**FIGURE 6:**
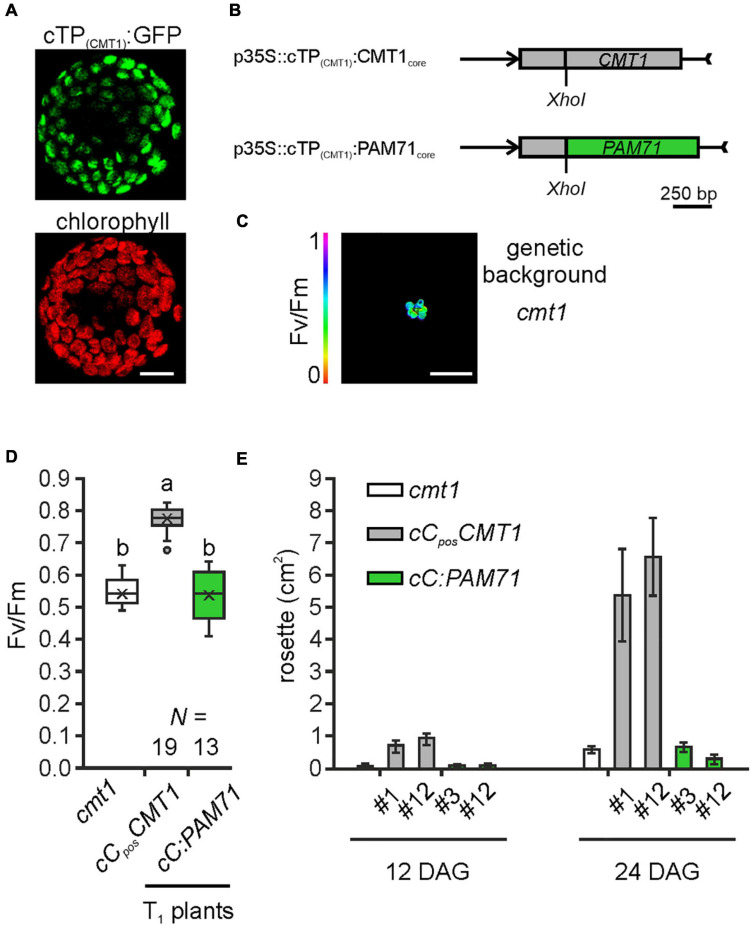
Experimental setup for gene replacement assay and analysis of individual transgenic lines in the *cmt1* genetic background. **(A)** Verification of the N-terminal CMT1 sequence as a chloroplast-targeting peptide. *Nicotiana benthamiana* leaves were infiltrated with the Agrobacterium strain containing p35S:cTP_(CMT1)_:GFP; protoplasts were isolated after 48 h and analyzed by confocal microscopy. Scale bar = 12.5 μm. **(B)** Schematic depiction of the chimeric constructs used for transformation of *cmt1.* **(C)** The *cmt1* mutant as genetic background. The maximum quantum yield of PSII (Fv/Fm) of a 4-week-old plant is shown. Scale bar = 1 cm. **(D)** Photosynthetic activity of BASTA resistant T_1_ transgenic plants in comparison to *cmt1*. The maximum quantum yield of PSII (Fv/Fm) of T_1_ plants is depicted as box plots representing the range of values, the exclusive median and the mean, indicated as x. The number of independent plant lines is given (N). Outliers are indicated as dots. Different letters indicate statistical significance according to ANOVA (*p* < 0.01, Tukey’s HSD test). **(E)** Quantification of growth phenotypes of the indicated genotypes. Leaf areas of selected transgenic lines in comparison to *cmt1* were determined from photographs taken 12 days after germination (DAG) and 24 days after germination (DAG). Mean values ± SD (*n* = 10) are shown.

Following the observation that overexpression of *CMT1* can complement the *pam71* phenotype, we also investigated the reverse configuration. First, we verified that the cTP of CMT1 was able to direct GFP into the chloroplast (Figure 6A). Next, a fusion construct consisting of the *cTP* of *CMT1* (designated as cC) and the core sequence of *PAM71* (Figure 6B) was assembled. This construct and a positive control (*cTP* of *CMT1* fused to the *CMT1* core sequence) were introduced into the *cmt1* genetic background, and several independent transgenic lines per construct were selected (Figure 6C). Comparison of the Fv/Fm values for both groups of plants revealed that, in contrast to *cC_*pos*_CMT1* lines, the Fv/Fm ratios in *cC:PAM71* lines resembled that of *cmt1* mutant plants (Figure 6D). Because subtle effects might not be detectable in the T1 generation, we selected two lines per group for closer inspection. The lines chosen exhibited the highest Fv/Fm values within their group in the respective T1 plant, carried a single-copy transgene insertion, and the magnitude of transgene expression was at least 10 times higher than the wild-type level (Supplementary Figures 5A,B). A re-examination of Fv/Fm values in subsequent generations revealed that, in both selected *cC:PAM71* lines, the Fv/Fm values were not significantly different from those of *cmt1* mutant plants, whereas Fv/Fm recovered to almost 0.8 in both positive control lines (Supplementary Figure 5C). In addition, growth of both *cC:PAM71* lines was markedly retarded, as in the case of *cmt1* mutant plants (Figure 6E). Taken together, these data showed that PAM71 cannot substitute CMT1 in the envelope membrane.

## Discussion

Our phylogenetic analysis indicates that eukaryotic and cyanobacterial members of the UPF0016 transporter family share a common ancestor, which is reflected in their functional conservation. A gene-replacement analysis was conducted in the Arabidopsis mutant *pam71* (Figure 3), which is characterized by diminished growth. This phenotype is readily discernible under standard cultivation conditions, and *pam71* is accessible to genetic manipulations resulting in inheritable traits. For our purposes, these features make it more suitable than systems in which the knock-out mutation generates a conditional phenotype. For instance, a conditional phenotype has been found in the yeast UPF0016 mutant *gdt1*, which is sensitive to high concentrations of Ca^2+^ (Demaegd et al., 2013). However, its growth rate is otherwise unaffected, i.e., similar to that of wild-type strains. It has been shown that sensitivity to excess Ca^2+^ can be suppressed by transient expression of TMEM165 (Demaegd et al., 2013).

As targets for our analysis, we selected the Synechocystis gene *MNX*, the *Arabidopsis* gene *CMT1* and the human gene *TMEM165.* Their protein products share with PAM71 the predicted topology of two clusters of three transmembrane domains, including the consensus motifs (Supplementary Figure 2). Variations at the N-terminus are assumed to be required for correct membrane targeting (Hoecker et al., 2017), and indeed the N-terminal portion of PAM71 is sufficient for targeting to chloroplasts (Figure 3A). Notably, a derivative of PAM71 lacking the chloroplast targeting signal peptide is incapable of complementing the *pam71* phenotype (Figures 3D, 4, 5B–D), indicating that the cTP is specifically required for correct targeting. Thus, the proteins MNX, CMT1 and TMEM165 could be successfully targeted to the thylakoid membrane of *pam71* plants by equipping them with the cTP of PAM71 (Figure 5A) and overexpressed from the respective transgenes (Figure 4D). We demonstrate that the paralog CMT1, as well as both orthologs SynMNX and HsTMEM165, are functional in Arabidopsis thylakoid membranes when introduced into the Arabidopsis *pam71* mutant background. These findings show that all four proteins have retained the ancestral function. Like PAM71, CMT1, MNX and TMEM165 are capable of transporting Mn^2+^ into the thylakoid lumen for efficient reconstitution of inorganic Mn clusters in photosystem II (Figures 5C,D)—as demonstrated by the recovery of photosystem II efficiency (Figure 4C), which eventually increased plant growth (Figure 4B). A strong proton gradient across the thylakoid membrane (Hohner et al., 2016; Pottosin and Shabala, 2016) is established upon illumination, which presumably energizes Mn^2+^ uptake into the acidic thylakoid lumen, in accordance with the current model of a cation/proton antiport mechanism for the UPF0016 protein family (Thines et al., 2020). In this respect, it is interesting to note that CMT1 can functionally replace PAM71 at the thylakoid membrane, but not *vice versa* (Figure 6 and Supplementary Figure 5). It is tempting to speculate that CMT1 has acquired additional features that allow it to cope with the conditions prevailing at the envelope membrane, where the proton gradient is much weaker (Hohner et al., 2016).

Our findings indicate that UPF0016 members function independently of additional factors and/or of the lipid composition of the membrane. It is particularly remarkable that the human ortholog TMEM165 can replace PAM71 in the thylakoid membrane, which is rich in monogalactosyldiacylglycerol and digalactosyldiacylglycerol (Li-Beisson et al., 2013; Rocha et al., 2018), in contrast to the Golgi membrane. TMEM165 (Foulquier et al., 2012; Potelle et al., 2016; Dulary et al., 2017; Lebredonchel et al., 2019; Foulquier and Legrand, 2020), like its plant counterpart PML3 (Hoecker et al., 2020; Yang et al., 2021), naturally resides in the Golgi membrane, where the Mn^2+^ imported into the Golgi lumen acts as an essential cofactor in the synthesis of N-glycans. In the present study an LC_MS/MS-based approach was employed to gain insight into the partitioning of overexpressed TMEM165, MNX, PAM71, and CMT1 between the envelope and thylakoid membranes, because quantitative proteomics studies on purified chloroplast membranes and their subfractions have been shown in the past to be an efficient method for this purpose (Ferro et al., 2010). The expected enrichment of marker proteins in the two membrane fractions allowed us to conclude that the method is reliable (Figure 5A). As observed in the past (Ferro et al., 2010), it was not possible to identify the endogenous PAM71 in wild-type samples (Supplementary Table 2); only in the overexpressor line *cP_*pos*_PAM71* could it be successfully identified (Figure 5A). Clearly, fractions of the overexpressed TMEM165, MNX, and PAM71 proteins were retained in the envelope membrane. However, at least in the case of PAM71, presumably non-functional (Figure 6).

UPF0016 proteins are of interest not only because of their conserved ability to facilitate Mn^2+^ transport, but also in terms of their evolutionary history—given that members have been identified across Archaea, Bacteria, and Eukaryota (Supplementary Figure 1). Small UPF0016 gene/protein families exist in plants, and in Arabidopsis the genes that code for both ER-localized proteins (PML4/PML5) are derived from a chromosomal duplication event in the progenitor of Arabidopsis (Hoecker et al., 2017)—indeed, these two share the highest similarity (Figure 2). This study suggests that the genes coding for the chloroplast-localized proteins CMT1 and PAM71 arose during the establishment of endosymbiosis, after the cyanobacterial precursor of chloroplasts contributed its gene copy to the eukaryotic host genome, which then gave rise to a gene duplication that is widely conserved in Viridiplantae (Figure 1). In green algae, the PAM71 homolog CGLD1 (conserved in the green lineage and diatome 1) maintained the structure and function of PSII, and contributed to its protection under photo-oxidative stress conditions (Xing et al., 2017). It is well established that genes of the cyanobacterial ancestor have been transferred to the nucleus, that many of the proteins they encode are rerouted to the chloroplast (Leister, 2003, 2016; Lee and Hwang, 2018) and that numerous proteins can functionally replace each other (Savidge et al., 2002; Sattler et al., 2003; Lv et al., 2009; Armbruster et al., 2013; Proctor et al., 2018; Yoon et al., 2019). Yet, what makes the evolution of the UPF0016 family particularly interesting is that it suggests all members of Eukaryota and Cyanobacteria (Figure 1) might have a common ancestor, which would explain why human TMEM165 can functionally substitute for PAM71 in chloroplast membranes. We suggest that plants received UPF0016 gene copies *via* two independent events. Although speculative, lateral gene transfer, perhaps involving Cyanobacteria, could be at the origin of subgroup one proteins, which are present in all Eukaryota, as there is no indication of an archaeal and/or protobacterial origin of this subgroup (Figure 1). In a second event, through cyanobacterial endosymbiotic gene transfer, subgroup two proteins were then introduced into the green lineage. We have not succeeded in tracing UPF0016 protein members to the last common ancestor of all cells, which is proposed to have lived about 3.8 billion years ago and to have had 355 protein clusters (Weiss et al., 2016, 2018). Thus, it is reasonable to assume that UPF0016 genes spread extensively by lateral gene transfer later in evolution.

It has also been suggested that PAM71 acquired an additional Ca^2+^ transport function (Wang et al., 2016; Frank et al., 2019). In this study, we have shown that the cyanobacterial MNX protein is functional in *pam71* plants and fully complements all aspects of the latter’s mutant phenotype (Figure 5). This enables us to conclude that only the Mn^2+^ transport activity of PAM71 plays a significant physiological role in chloroplasts. At the thylakoid membrane it can be replaced by its paralog (CMT1) and by its orthologs from *Zea mays* (Wang et al., 2020), cyanobacteria and human, yet there is no indication that PAM71 can function at the envelope membrane. A future challenge is to determine the molecular basis for the adaptation of CMT1 to the conditions prevailing in the envelope membrane of chloroplasts, and this study opens the door to further investigations of how far proteins can diverge without losing their ancient function.

## Data Availability Statement

The mass spectrometry proteomics data have been deposited at https://www.ebi.ac.uk/pride under the accession number PXD022763.

## Author Contributions

AS: conceptualization. NH, YH, SS, GM, and SBS: investigation and analysis of data. DL and AS: resources. SBS and AS: funding acquisition and writing—review and editing. NH and AS: writing—original draft. All authors contributed to manuscript revision, read, and approved the submitted version.

## Conflict of Interest

The authors declare that the research was conducted in the absence of any commercial or financial relationships that could be construed as a potential conflict of interest.

## Footnotes

1 https://blast.ncbi.nlm.nih.gov/Blast.cgi
