# Supplementary material for: Gene Replacement in Arabidopsis Reveals Manganese Transport as an Ancient Feature of Human, Plant and Cyanobacterial UPF0016 Proteins
Source: Front Plant Sci. 2021 Jun 14;12:697848. doi: 10.3389/fpls.2021.697848 (PMC8236900; doi:10.3389/fpls.2021.697848)
Supplement: Supplementary file 1 [file Data_Sheet_1.PDF]

Hoecker et al., 2021

Supplemental Figures and Tables

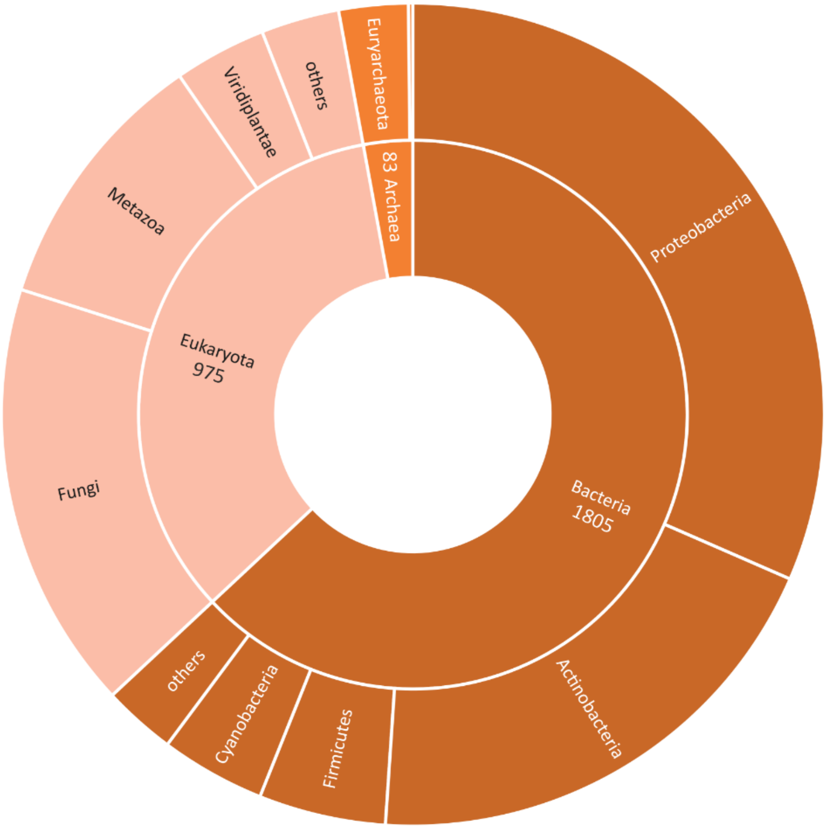

Figure S1. Distribution of UPF0016 sequences among Eukaryotes, Bacteria and Archaea. Data were retrieved from the protein family database at http://pfam.xfam.org/ accessed on 01. March 2021. The three superkingdoms including currently collected number of species within the Eukaryote, Bacteria and Archaea domains are shown. The eukaryotic domain is divided into kingdoms (Fungi, Metazoa, Viridiplantae and others) and bacterial and archaeal domains are divided into phyla.

```
cP-TMEM         MLSLNLSESLRIPFQNPRPPKSDFSSTSSSPSSSSRRCVSAYPIPIGFSVRNQYFSRCLT  60
cP-CMT1         MLSLNLSESLRIPFQNPRPPKSDFSSTSSSPSSSSRRCVSAYPIPIGFSVRNQYFSRCLT  60
cP-MNX          MLSLNLSESLRIPFQNPRPPKSDFSSTSSSPSSSSRRCVSAYPIPIGFSVRNQYFSRCLT  60
cP-PAM71        MLSLNLSESLRIPFQNPRPPKSDFSSTSSSPSSSSRRCVSAYPIPIGFSVRNQYFSRCLT  60
negPAM71        ------------------------------------------------------------  0

cP-TMEM         QLRRNESQQLGFRCFQRNDPACYLEAAAAPGNGRASAPRLLLLFLVPLLWAPAAVRAGPD  120
cP-CMT1         QLRRNESQQLGFRCFQRNDAACYLEGPFPYSLSIALVL----LSC---------------  101
cP-MNX          QLRRNESQQLGFRCFQRNDAACYLEKAESEEHDRNLDV----LVE---------------  101
cP-PAM71        QLRRNESQQLGFRCFQRNDAACYLEKAESEEHDRNLDV----LVE---------------  101
negPAM71        ----------------------MLEKAESEEHDRNLDV----LVE---------------  19
                                       **       .         *
                            - - - - - - - - - - - -
cP-TMEM         EDLSHRNKEPPAPAQ-QLQPQPVAVQGPEPARVEKIFTPAAPVHTNKEDPATQTNLGFIH  179
cP-CMT1         -GL------VFSLITF---------VKGGPS---------------SV-----LAAVAKS  125
cP-MNX          SSIAHSRREIQRVLMFLAVSGSVALLGTDPAFAASSI----PNVTQSLVTSFGDLGDILT  157
cP-PAM71        SSIAHSRREIQRVLMFLAVSGSVALLGTDPAFAASSI----PNVTQSLVTSFGDLGDISS  157
negPAM71        SSIAHSRREIQRVLMFLAVSGSVALLGTDPAFAASSI----PNVTQSLVTSFGDLGDISS  75
                 .:                          *:
                __________TM1__________          ________TM2_________
cP-TMEM         AFVAAISVIIVSELGDKTFFIAAIMAMRYNRLTVLAGAMLALGLMTCLSVLFGYATTVIP  239
cP-CMT1         GFTAAFSLIFVSEIGDKTFFIAALLAMQYEKTLVLLGSMGALSLMTILSVVIGKIFQSVP  185
cP-MNX          AFTAGLLLITVSELGDKTFFIAMILAMRYPRRWVLVGVVGGLAAMTILSVLMGQIFTFLP  217
cP-PAM71        GFASAFLLIFFSELGDKTFFIAALLAARNSAATVFVGTFGALGIMTIISVVLGRTFHYVD  217
negPAM71        GFASAFLLIFFSELGDKTFFIAALLAARNSAATVFVGTFGALGIMTIISVVLGRTFHYVD  135
                .*.:.: :* .**:******** ::* :     *: * . .*. ** :**::*     :
                _______________TM3________________
cP-TMEM         RVY-----------TYYVSTVLFAIFGIRMLREGLKMSPDEGQEELEEVQAELKKKDEEF  288
cP-CMT1         AQF-----QTTLPIGEYAAIALLMFFGLKSIKDAWDLPPVEAKNGEETGIELGEYSEAEE  240
cP-MNX          TRY-----------INYAEVALFLIFGTKLLWDARR---IKATANLEE------MEDAEK  257
cP-PAM71        EVLPFRFGGTDLPIDDIAAVCLLVYFGVSTLLDAVS---DEGKADEEQ------K-EAEL  267
negPAM71        EVLPFRFGGTDLPIDDIAAVCLLVYFGVSTLLDAVS---DEGKADEEQ------K-EAEL  185
                                 .   *:  **   : :.      :.    *         : *
                                          ____________TM4_____________
cP-TMEM         QRTKLLNGPGDVETGTSITVPQKKWLHFISPIFVQALTLTFLAEWGDRSQLTTIVLAARE  348
cP-CMT1         LV---KEK-------------ASKKLTNPLEILWKSFSLVFFAEWGDRSMLATVALGAAQ  284
cP-MNX          AIAS-GEK-------------KLKIVPRGWGIVVESFALTFVAEWGDRTQIATIALAASN  303
cP-PAM71        AVSE-LSG-------------NGAGIVAAANTIISTFALVFVAEWGDKSFFSTIALAAAS  313
negPAM71        AVSE-LSG-------------NGAGIVAAANTIISTFALVFVAEWGDKSFFSTIALAAAS  231
                      .                  :      . .:::*.*.*****:: ::*:.*.* .
                  _________TM5_________          _________TM6_________
cP-TMEM         DPYGVAVGGTVGHCLCTGLAVIGGRMIAQKISVRTVTIIGGIVFLAFAFSALFISPDSGF  408
cP-CMT1         SPLGVASGAIAGHLVATVLAIMGGAFLANYISEKLVGYVGGALFLVFAAATFFGVF----  340
cP-MNX          NAWGVSAGAILGHTICAVIAVMGGKFVAGRISEKTVTLIGGLLFYLFAVVSWWTKIA---  360
cP-PAM71        SPLGVIAGALAGHGAATLLAVLGGSLLGNFLSEKAIAYVGGVLFLVFAAVTVAEIVT---  370
negPAM71        SPLGVIAGALAGHGAATLLAVLGGSLLGNFLSEKAIAYVGGVLFLVFAAVTVAEIVT---  288
                .  **  *.  **  .: :*::** ::.  :* : :  :** :*  **  :
```

Figure S2. Sequence alignment of chimeric proteins containing the chloroplast-targeting peptide (cTP) of PAM71.

The alignment was generated using Clustal Omega (version 1.2.4). The sequences of the cTP and the PAM71 core protein are shown in green, the sequence of the CMT1 core protein is shown in black, the sequence of the TMEM165 protein is shown in orange, and the sequence of the MNX protein is shown in blue. The two conserved E-x-G-D-(KR)-(TS) motifs are highlighted in red, six transmembrane domains are indicated, and an additional putative transmembrane domain is indicated by a dashed line. Asterisk (*) indicates fully conserved residues; colon (:) indicates conservation between groups of strongly similar properties; and a period (.) indicates conservation between groups of weakly similar properties.

A

plant line	T_1 plant Fv/Fm	T_2 plants segregation (+ : -)	assumption 3:1 model
$cP_{pos}PAM71$ #17	0,82	27 : 9	$\chi^2 = 0$
$cP_{pos}PAM71$ #25	0,83	30 : 6	$\chi^2 = 1{,}33$
$cP_{neg}PAM71$ #2	0,58	25 : 10	$\chi^2 = 0{,}24$
$cP_{neg}PAM71$ #8	0,57	27 : 8	$\chi^2 = 0{,}09$
cP:CMT1 #16	0,82	23 : 13	$\chi^2 = 2{,}37$
cP:CMT1 #27	0,82	28 : 8	$\chi^2 = 0{,}15$
cP:TMEM165 #13	0,78	28 : 8	$\chi^2 = 0{,}15$
cP:TMEM165 #39	0,78	30 : 6	$\chi^2 = 1{,}33$
cP:MNX #1	0,79	30 : 6	$\chi^2 = 1{,}33$
cP:MNX #34	0,80	28 : 8	$\chi^2 = 0{,}15$

B

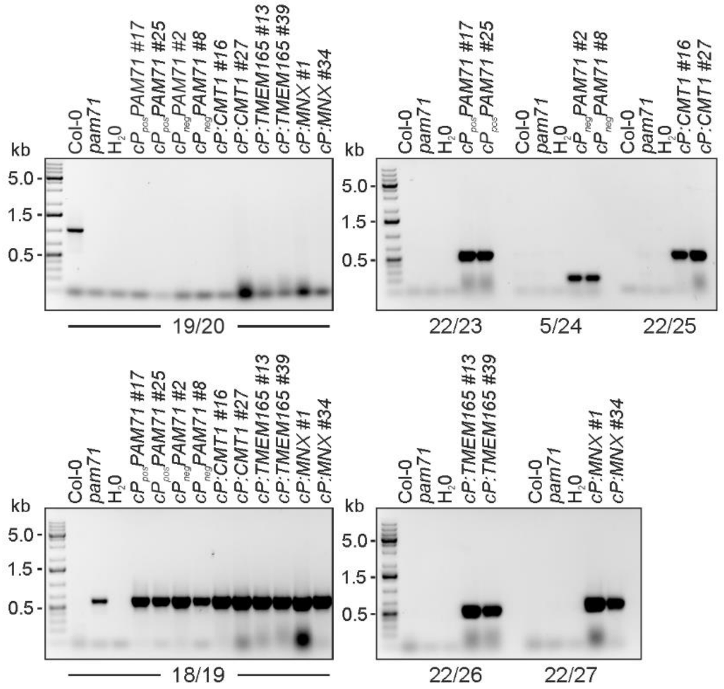

Figure S3. Selection of individual transgenic lines with *pam71* genetic background.

(A) The maximum quantum yield of PSII (Fv/Fm) of selected T_1 plants is shown in the left panel. Selected T_1 plants were allowed to self-pollinate and the presence (+) and absence (-) of transgenes in the following generation (T_2) determined (middle panel). A $\chi 2$ test was applied to judge if the null-hypothesis of a 3:1 segregation can be rejected or not (right panel). For values below 3,84 (degree of freedom: 1) the null-hypothesis cannot be rejected, which means it probably applies (α = 0,05).

(B) The indicated transgenic plant lines were subjected to PCR analysis using three primer combinations amplifying the wild-type allele (19/20, Table S1), the *pam71* mutant allele

(18/19, Table S1) and the specific constructs (right panels, for primer sequences see Table S1) for verification. The primer combinations (given in the right panels) were used for the analysis in (A).

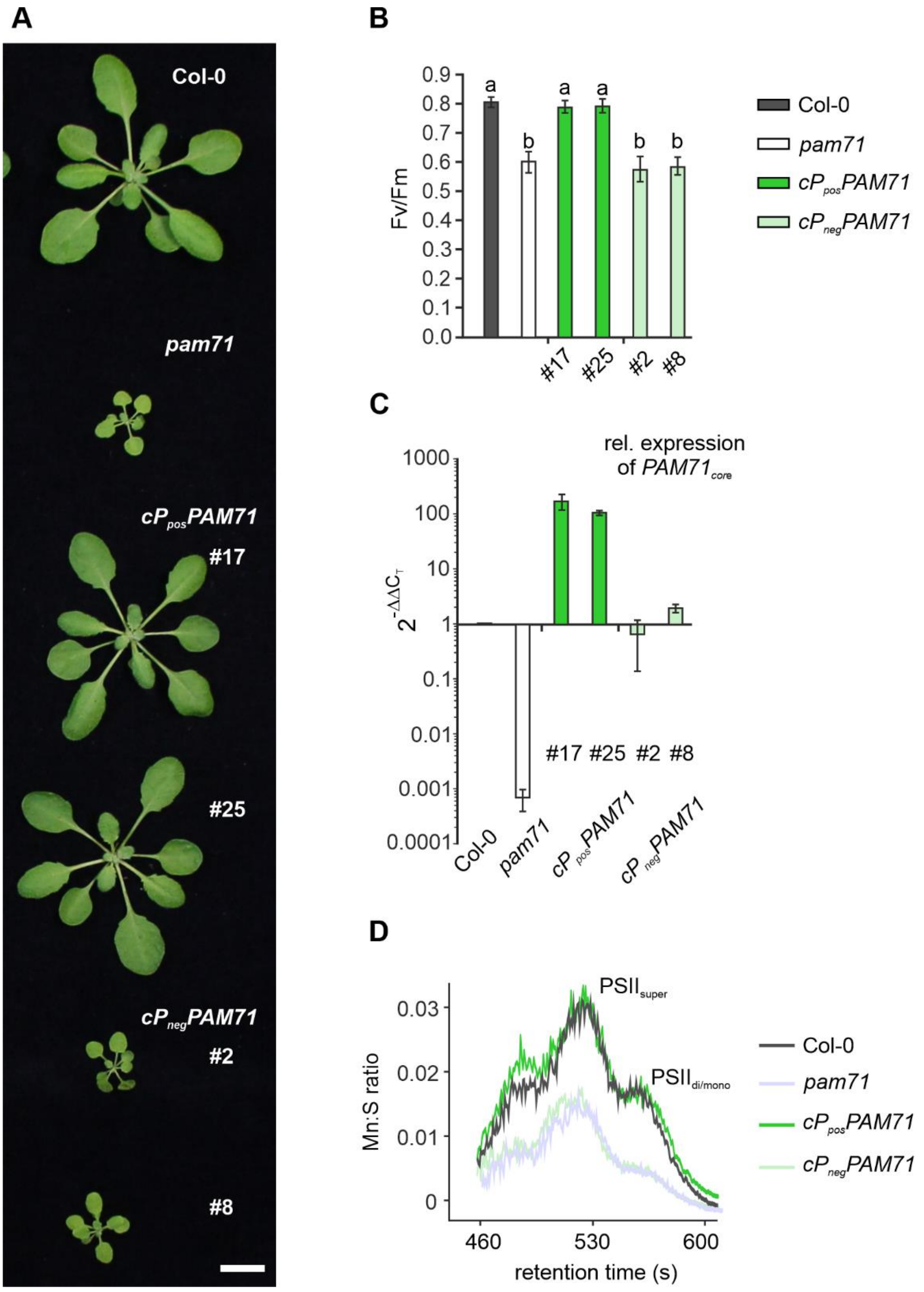

Figure S4. Comparison of *cP$_{pos}$PAM71* and *cP$_{neg}$PAM71* to wild-type and *pam71* plants.

(A) Rosette phenotype of the indicated genotype. Plants were grown for 3,5 weeks in a 12 h-12 h light-dark cycle. Scale bar = 1 cm.

(B) Photosynthetic activity of the indicated genotypes. The maximum quantum yield of PSII (Fv/Fm) is shown as mean values ± SD (n ≥ 17). Plants were grown for 4 weeks in a 12 h-

12 h light-dark cycle at 100 µmol photons m^{-2} s^{-1}. Different letters indicate statistical significance according to ANOVA ($p < 0.05$, Tukey's HSD test).

(C) Expression analysis of *PAM71*$_{core}$. Quantitative real-time PCR (qRT-PCR) was performed using primer combination qRT_PAM71_fwd/qRT_PAM71_rev (Table S1). Expression levels are relative values based on the expression levels in Col-0 (=1), and *Actin2* as reference gene. Mean values ± SD are based on three biological replicates.

(D) Quantification of the Mn:S stochiometric ratios in the indicated genotypes. Mn:S ratios are shown in fractionated photosynthetic complexes. PSII = Photosystem II; different assembly states are indicated. Analysis was performed with two independent experiments.

A

plant line	T_1 plant Fv/Fm	T_2 plants segregation (+ : -)	assumption 3:1 model
cmt1	--	--	--
cC$_{pos}$CMT1 #1	0,81	27 : 8	χ^2 = 0,09
cC$_{pos}$CMT1 #12	0,80	24 : 10	χ^2 = 0,24
cC:PAM71 #3	0,62	23 : 13	χ^2 = 2,37
cC:PAM71 #12	0,57	25 : 11	χ^2 = 0,59

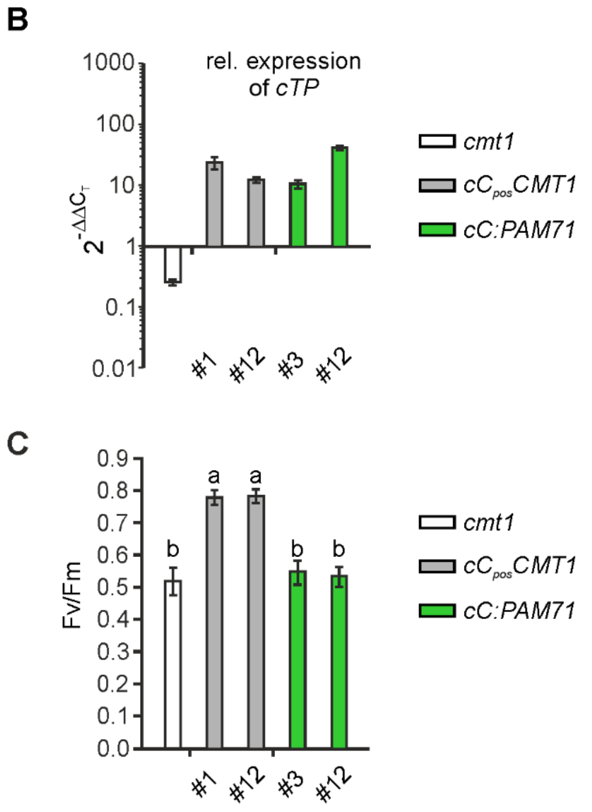

Figure S5. Selection of individual transgenic lines with *cmt1* as background.

(A) The maximum quantum yield of PSII (Fv/Fm) of selected T_1 plants in comparison to *cmt1* is shown in the left panel. Selected T_1 plants were allowed to self-pollinate and the presence (+) and absence (-) of transgenes in the following generation (T_2) determined (middle panel). A $\chi 2$ test was applied to judge if the null-hypothesis of a 3:1 segregation can be rejected or not (right panel). For values below 3,84 (degree of freedom: 1) the null-hypothesis cannot be rejected, which means it probably applies (α = 0,05).

(B) Expression analysis in transgenic lines in comparison to *cmt1*. Quantitative real-time PCR (qRT-PCR) was performed using primer combination

qRT_CMT1cTP_fwd/qRT_CMT1cTP_rev (Table S1) for expression of cTP_{CMT1}. Expression levels are relative values based on the expression levels in Col-0 (=1), and Actin2 as reference gene. Mean values ± SD are based on three biological replicates.

(C) Photosynthetic activity of transgenic lines in comparison to *cmt1.* The maximum quantum yield of PSII (Fv/Fm) of selected transgenic lines is shown as mean values ± SD ($n \geq 17$) in comparison to *cmt1* plants. Plants were grown for 4 weeks in a 12 h-12 h light-dark cycle at 100 µmol photons m-2 s-1. Different letters indicate statistical significance according to ANOVA ($p < 0.05$, Tukey's HSD test).

Table S1: Oligonucleotides used in this study. Vector sequences and restriction sites are underlined. Primers 10 to 15 were used in Gibson assembly (GA) cloning, GK-LB is the abbreviation for Gabi-Kat-Left-Border, GT stands for genotyping.

	Primer name	Sequence (5'-3')	Application
1	PAM71cTP_F	CAC CAT GCT AAG TTT GAA TCT CTC G	Generation of chimeric constructs
2	PAM71cTP_R	TTT CTC GAG GTA ACA AGC CGC	
3	PAM71core-F	TTT CTC GAG AAA GCT GAG TCT GAA G	
4	PAM71core-R	TTA CGT AAC GAT CTC AGC CAC C	
5	PAM71neg-F	CAC CAT GCT CGA GAA AGC TG	
6	CMT1core-F	AAC CTC GAG GGG CCA TTT C	
7	CMT1core-R	TCA GAA CAC TCC AAA GAA TGT GG	
8	TMEM165-F	TTT CTC GAG GCG GCC GCG GCT CCA GGG AAC GGC	
9	TMEM165-R	TTA AAA ACC AGA ATC AGG GCT TAT	
10	GA_PAM71N-F	CAT TAC AAT TTA CTA TTC TAG TCG AAT GCT AAG TTT GAA TCT CTC	
11	GA_PAM71N-R	TAA AAG CGG TCA GAA TAT CTC CAA GGT CTC C	
12	GA_MNX-F	CCT TGG AGA TAT TCT GAC CGC TTT TAC TGC C	
13	GA_MNX-R	ATT TTT GCG GAC TCT AGC ATG GCC GCT ATG CAA TCT TGG TCC AC	
14	GA_pB2GW7-F	CGG CCA TGC TAG AGT CCG CAA AAA TCA C	
15	GA_pB2GW7-R	TCG ACT AGA ATA GTA AAT TGT AAT G	
16	CMT1cTP_F	CAC CAT GAA GCT CAC AAG CTT G	
17	CMT1cTP_R	TTT CTC GAG GCT TTC TGA TGA AGT G	
18	GK-LB	ATA TTG ACC ATC ATA CTC ATT GC	Genotyping
19	1g64150-F	AAT TCT CCT GAA ACT TGG AAG ACA	
20	1g64150-R	TTC GGT CTT TAG AAC ACA CTC TCT	
21	GT-CMT1cTP-F	CAG ATG CTG GTG TTG GAT C	
22	GT-PAM71cTP-F	GTC TCC GCT TAT CCA ATT CC	
23	GT-PAM71-R1	GAC ATA GTG GAA AGT GCG TC	
24	GT-PAM71-R2	CAA GGT CTC CAA AAG AAG TGA CA	
25	GT-CMT1-R	CAC CAT TCT TGG CTT CCA C	
26	GT-TMEM165-R	CAT GTC ATT AGT CCC AAG GC	
27	GT-MNX-R	CTG CAT CTT CCA TTT CCT CC	
28	qRT_actin_fwd	CTC TTT CTT TCC AAG CTC ATA AAA AAT G	qRT-PCR
29	qRT_actin_rev	CAG CAC AAT ACC GGT TGT ACG AC	
30	qRT_PAM71cTP_fwd	GTC GCT TCG CAT TCC ATT CC	
31	qRT_PAM71cTP_rev	TAA CAA GCC GCA TCG TTT CG	
32	qRT_PAM71_fwd	CAA GAA GAG AGA TTC AGA GAG TTC	
33	qRT_PAM71_rev	GAA ACA GAA CTC CTC CGA CAT AAG	
34	qRT_CMT1_fwd	TAA TTC CAC AGC AAC AGC AG	
35	qRT_CMT1_rev	ATT TTC CCC AGA GCA AAT CC	

Table S2. Proteomic analysis of envelope and thylakoid membrane fractions isolated from wild-type (Col-0) and *cP_{neg}PAM71* #8 plants.

		**Col-0**		***cP$_{neg}$PAM71***	
**UniProt ID**	**protein name**	**thylakoid (%)**	**envelope (%)**	**thylakoid (%)**	**envelope (%)**
**Q94AX5**	PAM71	n.d.	n.d.	n.d.	n.d.
**Q9T0H9**	CMT1 (Arabidopsis)	n.d.	100,00	19,92	80,08
**Q9HC07**	TMEM165 (human)	n.d.	n.d.	n.d.	n.d.
**P52876**	MNX (Synechocystis)	n.d.	n.d.	n.d.	n.d.
	**envelope marker:**				
**Q9ZTZ7**	K(+) efflux antiporter 1	12,88	87,12	25,66	74,34
**O65272**	K(+) efflux antiporter 2	14,73	85,27	5,60	94,40
**Q9LXV3**	Dicarboxylate transporter 1	10,40	89,60	31,60	68,40
**Q9FMF7**	Dicarboxylate transporter 2.1	7,31	92,69	6,28	93,72
**Q9FMF8**	Dicarboxylate transporter 2.2	n.d.	100,00	n.d.	100,00
**Q9ZSR7**	Triose phosphate/phosphate translocator	20,88	79,12	6,61	93,39
**Q8RXN3**	Phosphoenolpyruvate/phosphate translocator 1	16,72	83,28	23,05	76,95
**Q9SZC9**	Copper-transporting ATPase PAA1	n.d.	100,00	n.d.	100,00
	**thylakoid marker:**				
**Q9SHR7**	Chlorophyll a-b binding protein 2.1	100,00	n.d.	n.d.	n.d.
**P83755**	Photosystem II protein D1	84,53	15,47	62,95	37,05
**P56761**	Photosystem II protein D2	82,97	17,03	57,29	42,71
**P56778**	Photosystem II CP43 reaction center protein	86,43	13,57	60,42	39,58
**P56777**	Photosystem II CP47 reaction center protein	91,87	8,13	82,27	17,73
**B9DFX7**	Copper-transporting ATPase PAA2	64,83	35,17	36,16	63,84
**Q9M0Z3**	K(+) efflux antiporter 3	73,62	26,38	63,89	36,11

Table S3. Proteomic analysis of thylakoid and envelope membrane fractions isolated from *cP$_{pos}$PAM71* #25 plants. Experiments were derived from three independent grown batches of plants.

cP$_{pos}$PAM71		**experiment 1**		**experiment 2**		**experiment 3**	
**UniProt ID**	**protein name**	**thylakoid (%)**	**envelope (%)**	**thylakoid (%)**	**envelope (%)**	**thylakoid (%)**	**envelope (%)**
**Q94AX5**	PAM71	51,96	48,04	50,48	49,52	33,13	66,87
**Q9T0H9**	CMT1 (Arabidopsis)	36,52	63,48	6,92	93,08	15,62	84,38
**Q9HC07**	TMEM165 (human)	n.d.	n.d.	n.d.	n.d.	n.d.	n.d.
**P52876**	MNX (Synechocystis)	n.d.	n.d.	n.d.	n.d.	n.d.	n.d.
	**envelope marker:**						
**Q9ZTZ7**	K(+) efflux antiporter 1	15,18	84,82	4,60	95,40	6,76	93,24
**O65272**	K(+) efflux antiporter 2	8,95	91,05	1,46	98,54	2,09	97,91
**Q9LXV3**	Dicarboxylate transporter 1	4,93	95,07	8,61	91,39	12,37	87,63
**Q9FMF7**	Dicarboxylate transporter 2.1	8,43	91,57	5,16	94,84	6,41	93,59
**Q9FMF8**	Dicarboxylate transporter 2.2	n.d.	100,00	n.d.	100,00	n.d.	100,00
**Q9ZSR7**	Triose phosphate/phosphate translocator	4,86	95,14	0,73	99,27	25,73	74,27
**Q8RXN3**	Phosphoenolpyruvate/phosphate translocator 1	8,15	91,85	19,25	80,75	23,85	76,15
**Q9SZC9**	Copper-transporting ATPase PAA1	11,44	88,56	n.d.	100,00	n.d.	100,00
	**thylakoid marker:**						
**Q9SHR7**	Chlorophyll a-b binding protein 2.1	100,00	n.d.	100,00	n.d.	100,00	n.d.
**P83755**	Photosystem II protein D1	76,18	23,82	78,25	21,75	67,08	32,92
**P56761**	Photosystem II protein D2	76,36	23,64	66,97	33,03	64,04	35,96
**P56778**	Photosystem II CP43 reaction center protein	68,99	31,01	60,89	39,11	63,50	36,50
**P56777**	Photosystem II CP47 reaction center protein	85,02	14,98	78,43	21,57	67,24	32,76
**B9DFX7**	Copper-transporting ATPase PAA2	46,39	53,61	n.d.	100,00	14,29	85,71
**Q9M0Z3**	K(+) efflux antiporter 3	100,00	n.d.	n.d.	100,00	n.d.	100,00

Table S4. Proteomic analysis of thylakoid and envelope membrane fractions isolated from *cP:MNX* #1 plants. Experiments were derived from three independent grown batches of plants.

cP:MNX		**experiment 1**		**experiment 2**		**experiment 3**	
**UniProt ID**	**protein name**	**thylakoid (%)**	**envelope (%)**	**thylakoid (%)**	**envelope (%)**	**thylakoid (%)**	**envelope (%)**
**Q94AX5**	PAM71	n.d.	n.d.	n.d.	n.d.	n.d.	n.d.
**Q9T0H9**	CMT1 (Arabidopsis)	22,63	77,37	21,29	78,71	9,98	90,02
**Q9HC07**	TMEM165 (human)	n.d.	n.d.	n.d.	n.d.	n.d.	n.d.
**P52876**	MNX (Synechocystis)	24,46	75,54	44,77	55,23	59,05	40,95
	**envelope marker:**						
**Q9ZTZ7**	K(+) efflux antiporter 1	16,62	83,38	23,43	76,57	8,26	91,74
**O65272**	K(+) efflux antiporter 2	6,94	93,06	9,43	90,57	5,08	94,92
**Q9LXV3**	Dicarboxylate transporter 1	4,21	95,79	16,17	83,83	15,25	84,75
**Q9FMF7**	Dicarboxylate transporter 2.1	9,04	90,96	15,92	84,08	2,52	97,48
**Q9FMF8**	Dicarboxylate transporter 2.2	n.d.	100,00	n.d.	100,00	n.d.	100,00
**Q9ZSR7**	Triose phosphate/phosphate translocator	4,69	95,31	17,18	82,82	7,17	92,83
**Q8RXN3**	Phosphoenolpyruvate/phosphate translocator 1	11,35	88,65	24,44	75,56	22,76	77,24
**Q9SZC9**	Copper-transporting ATPase PAA1	n.d.	100,00	n.d.	n.d.	29,76	70,24
	**thylakoid marker:**						
**Q9SHR7**	Chlorophyll a-b binding protein 2.1	79,55	20,45	69,08	30,92	90,17	9,83
**P83755**	Photosystem II protein D1	69,07	30,93	71,18	28,82	57,92	42,08
**P56761**	Photosystem II protein D2	69,99	30,01	64,70	35,30	56,54	43,46
**P56778**	Photosystem II CP43 reaction center protein	73,88	26,12	67,37	32,63	59,29	40,71
**P56777**	Photosystem II CP47 reaction center protein	92,50	7,50	80,17	19,83	58,38	41,62
**B9DFX7**	Copper-transporting ATPase PAA2	47,96	52,04	48,57	51,43	27,46	72,54
**Q9M0Z3**	K(+) efflux antiporter 3	68,93	31,07	23,36	76,64	n.d.	100,00

Table S5. Proteomic analysis of envelope and thylakoid membrane fractions isolated from c*P:TMEM165* #39 plants. Experiments were derived from three independent grown batches of plants.

**c*P:TMEM165***		**experiment 1**		**experiment 2**		**experiment 3**	
**UniProt ID**	**protein name**	**thylakoid (%)**	**envelope (%)**	**thylakoid (%)**	**envelope (%)**	**thylakoid (%)**	**envelope (%)**
**Q94AX5**	PAM71	n.d.	n.d.	n.d.	n.d.	n.d.	n.d.
**Q9T0H9**	CMT1 (Arabidopsis)	30,77	69,23	17,96	82,04	18,62	81,38
**Q9HC07**	TMEM165 (human)	16,97	83,03	30,40	69,60	16,87	83,13
**P52876**	MNX (Synechocystis)	n.d.	n.d.	n.d.	n.d.	n.d.	n.d.
	**envelope marker:**						
**Q9ZTZ7**	K(+) efflux antiporter 1	12,66	87,34	15,07	84,93	12,74	87,26
**O65272**	K(+) efflux antiporter 2	6,59	93,41	6,83	93,17	6,87	93,13
**Q9LXV3**	Dicarboxylate transporter 1	35,29	64,71	3,25	96,75	22,15	77,85
**Q9FMF7**	Dicarboxylate transporter 2.1	5,88	94,12	31,21	68,79	2,33	97,67
**Q9FMF8**	Dicarboxylate transporter 2.2	10,80	89,20	n.d.	100,00	n.d.	100,00
**Q9ZSR7**	Triose phosphate/phosphate translocator	32,10	67,90	25,84	74,16	13,19	86,81
**Q8RXN3**	Phosphoenolpyruvate/phosphate translocator 1	12,30	87,70	20,73	79,27	22,26	77,74
**Q9SZC9**	Copper-transporting ATPase PAA1	20,02	79,98	n.d.	100,00	n.d.	100,00
	**thylakoid marker:**						
**Q9SHR7**	Chlorophyll a-b binding protein 2.1	100,00	n.d.	100,00	n.d.	100,00	n.d.
**P83755**	Photosystem II protein D1	68,46	31,54	81,18	18,82	66,24	33,76
**P56761**	Photosystem II protein D2	73,07	26,93	75,92	24,08	63,46	36,54
**P56778**	Photosystem II CP43 reaction center protein	79,22	20,78	74,47	25,53	73,39	26,61
**P56777**	Photosystem II CP47 reaction center protein	93,74	6,26	83,35	16,65	68,08	31,92
**B9DFX7**	Copper-transporting ATPase PAA2	41,16	58,84	60,33	39,67	46,31	53,69
**Q9M0Z3**	K(+) efflux antiporter 3	40,76	59,24	64,83	35,17	23,38	76,62

Table S6. Proteomic analysis of envelope and thylakoid membrane fractions isolated from c*P:CMT1* #27 plants. Experiments were derived from three independent grown batches of plants.

**c*P:CMT1***		**experiment 1**		**experiment 2**		**experiment 3**	
**UniProt ID**	**protein name**	**thylakoid (%)**	**envelope (%)**	**thylakoid (%)**	**envelope (%)**	**thylakoid (%)**	**envelope (%)**
**Q94AX5**	PAM71	n.d.	n.d.	n.d.	n.d.	n.d.	n.d.
**Q9T0H9**	CMT1 (Arabidopsis)	55,61	44,39	45,50	54,50	11,16	88,84
**Q9HC07**	TMEM165 (human)	n.d.	n.d.	n.d.	n.d.	n.d.	n.d.
**P52876**	MNX (Synechocystis)	n.d.	n.d.	n.d.	n.d.	n.d.	n.d.
	**envelope marker:**						
**Q9ZTZ7**	K(+) efflux antiporter 1	15,20	84,80	18,48	81,52	3,71	96,29
**O65272**	K(+) efflux antiporter 2	8,01	91,99	9,83	90,17	2,73	97,27
**Q9LXV3**	Dicarboxylate transporter 1	9,49	90,51	24,95	75,05	10,64	89,36
**Q9FMF7**	Dicarboxylate transporter 2.1	20,36	79,64	24,30	75,70	8,24	91,76
**Q9FMF8**	Dicarboxylate transporter 2.2	n.d.	100,00	n.d.	n.d.	n.d.	100,00
**Q9ZSR7**	Triose phosphate/phosphate translocator	24,12	75,88	49,28	50,72	12,27	87,73
**Q8RXN3**	Phosphoenolpyruvate/phosphate translocator 1	23,96	76,04	41,97	58,03	10,75	89,25
**Q9SZC9**	Copper-transporting ATPase PAA1	n.d.	100,00	22,07	77,93	n.d.	100,00
	**thylakoid marker:**						
**Q9SHR7**	Chlorophyll a-b binding protein 2.1	96,66	3,34	79,86	20,14	88,01	11,99
**P83755**	Photosystem II protein D1	67,53	32,47	91,97	8,03	64,80	35,20
**P56761**	Photosystem II protein D2	69,32	30,68	90,42	9,58	73,55	26,45
**P56778**	Photosystem II CP43 reaction center protein	83,18	16,82	91,81	8,19	83,50	16,50
**P56777**	Photosystem II CP47 reaction center protein	87,81	12,19	84,22	15,78	87,80	12,20
**B9DFX7**	Copper-transporting ATPase PAA2	65,93	34,07	78,44	21,56	n.d.	n.d.
**Q9M0Z3**	K(+) efflux antiporter 3	57,18	42,82	91,25	8,75	72,41	27,59

Table S7. Proteomic analysis of envelope and thylakoid membrane fractions isolated from c*P:CMT1* #27 plants, which were grown supplementary to experiments 1-3. Median and mean absolute deviation (MAD) were calculated from data given in Tables S2 to S7.

		**experiment 4**		**Median ± MAD of exp 1-4 comprising genotype c*P:CMT1* N = 4**		**Median ± MAD of exp 1-3 comprising genotypes *cP$_{pos}$:PAM71*, *cP:TMEM165*, *cP:MNX*, and Col-0 and *cP$_{neg}$PAM71* N = 11**	
**UniProt ID**	**protein name**	**thylakoid (%)**	**envelope (%)**	**thylakoid (%)**	**envelope (%)**	**thylakoid (%)**	**envelope (%)**
**Q9T0H9**	CMT1 (Arabidopsis)	34,09	65,91	39,80 ±13,97	60,20 ±13,97	18,62 ±7,37	81,38 ±7,37
	**envelope marker:**						
**Q9ZTZ7**	K(+) efflux antiporter 1	15,80	84,20	15,50 ±4,79	84,50 ±4,79	12,88 ±4,73	87,12 ±4,73
**O65272**	K(+) efflux antiporter 2	9,18	90,82	8,60 ±2,35	91,40 ±2,35	6,83 ±2,38	93,17 ±2,38
**Q9LXV3**	Dicarboxylate transporter 1	12,30	87,70	11,47 ±5,30	88,53 ±5,30	12,37 ±8,33	87,63 ±8,33
**Q9FMF7**	Dicarboxylate transporter 2.1	13,90	86,10	17,13 ±5,63	82,87 ±5,63	6,41 ±5,25	93,59 ±5,25
**Q9FMF8**	Dicarboxylate transporter 2.2	n.d.	100,00	n.d.	100 ±0	0 ±1,79	100 ±1,79
**Q9ZSR7**	Triose phosphate/phosphate translocator	32,08	67,92	28,10 ±11,24	71,90 ±11,24	13,19 ±8,99	86,81 ±8,99
**Q8RXN3**	Phosphoenolpyruvate/phosphate translocator 1	34,22	65,78	29,09 ±10,37	70,91 ±10,37	20,73 ±4,72	79,27 ±4,72
**Q9SZC9**	Copper-transporting ATPase PAA1	n.d.	100,00	0 ±8,28	100 ±8,28	10,01 ±12,45	89,99 ±12,45
